# Metal–Organic Frameworks as Potential Agents for Extraction and Delivery of Pesticides and Agrochemicals

**DOI:** 10.1021/acsomega.2c05978

**Published:** 2022-12-08

**Authors:** Lila A. M. Mahmoud, Roberta A. dos Reis, Xianfeng Chen, Valeska P. Ting, Sanjit Nayak

**Affiliations:** † School of Chemistry and Biosciences, University of Bradford, Bradford BD7 1DP, United Kingdom; ‡ School of Pharmacy, Al-Zaytoonah University of Jordan, Amman 11733, Jordan; § Centro de Ciências Naturais e Humanas, Universidade Federal do ABC, Santo André, SP 09210, Brazil; ∥ School of Engineering, Institute for Bioengineering, The University of Edinburgh, Edinburgh EH9 3JL, United Kingdom; ⊥ Bristol Composites Institute, Department of Mechanical Engineering, University of Bristol, Bristol BS8 1TR, United Kingdom

## Abstract

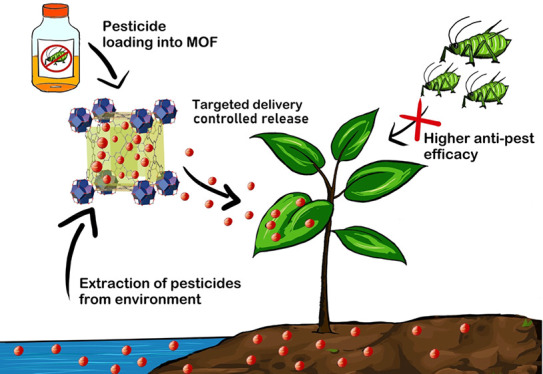

Pesticide contamination is a global issue, affecting nearly 44% of the global farming population, and disproportionately affecting farmers and agricultural workers in developing countries. Despite this, global pesticide usage is on the rise, with the growing demand of global food production with increasing population. Different types of porous materials, such as carbon and zeolites, have been explored for the remediation of pesticides from the environment. However, there are some limitations with these materials, especially due to lack of functional groups and relatively modest surface areas. In this regard, metal–organic frameworks (MOFs) provide us with a better alternative to conventionally used porous materials due to their versatile and highly porous structure. Recently, a number of MOFs have been studied for the extraction of pesticides from the environment as well as for targeted and controlled release of agrochemicals. Different types of pesticides and conditions have been investigated, and MOFs have proved their potential in agricultural applications. In this review, the latest studies on delivery and extraction of pesticides using MOFs are systematically reviewed, along with some recent studies on greener ways of pest control through the slow release of chemical compounds from MOF composites. Finally, we present our insights into the key issues concerning the development and translational applications of using MOFs for targeted delivery and pesticide control.

## 1 Introduction

Pesticides are a class of chemicals that prevent the unwanted growth and infestation of pests. Pesticides can be classified according to their (i) chemical structures, (ii) target organism, and (iii) their mode of action. By referring to their chemical compositions, one of the most commonly used classes of pesticides is organophosphates, a group of compounds with a central phosphate atom,^1^ which includes the common insecticides diazinon, chlorpyrifos, malathion,^2^ fenitrothion,^3^ as well as the herbicide glyphosate.^4^ Other groups include chlorophenoxy herbicides, which include 2,4-dichlorophenoxyacetic acid (2,4-D) and 2-methyl-4-chlorophenoxyacetic acid (MCPA)^5^ and carbamates, formally synthesized from carbamic acid,^6^ pyrethroids, which have structures analogous to the naturally occurring pyrethrins,^7^ and neonicotinoids, with a similar structure to nicotine.^8^

On the basis of their target pests, pesticides are classified into fungicides, insecticides, herbicides, rodenticides, and so on. Common antifungal agents that belong to the triazole and imidazole family are commonly used, like epoxiconazole.^9^ Insecticides, which are chemicals intended to kill insects, include the compound dichlorodiphenyltrichloroethane (DDT), which was proven to have significant detrimental environmental and health risks^10^ and was widely replaced by chlorpyrifos. Herbicides which make up 50% of total pesticides used include the widely used broad-leaf weed controlling agent glyphosate.^11^

Based on their modes of action, pesticides have various mechanisms; for example, organophosphate and carbamate insecticides act as acetylcholine esterase inhibitors, preventing the breakdown of acetylcholine and results in the overstimulation of the nervous system.^12^ Other insecticides act on voltage-gated ionic channels^13^ and GABA receptors.^14^ Herbicides act by inhibiting essential metabolic plant-specific pathway processes, and they can be grouped by their target mechanism, like auxin receptor herbicides^15^ and photosystem II inhibitors.^16^

The application of pesticides can help to decrease yield loss as a result of weeds,^17^ improve the quality of harvest by protecting against pathogenic diseases,^18^ and reduce the loss of perishable crops as fruits and vegetables during storage and transport.^19^ Pesticides also help to reduce fungal contaminants such as aflatoxins, a known liver carcinogen.^18−20^ In addition, pesticides provide secondary benefits to livestock,^21^ overall economic growth,^22^ and ensure food security.^11^ For example, in the U.S., it was estimated that the loss of crops would range from 20% for corn to 80% for peanuts without the use of pesticides.^23^ A more recent study estimated that the potential loss of winter wheat due to weeds is approximately 10.5 billion kg with an approximate value of 2.19 billion U.S. dollars for the United States and Canada.^24^

Despite their highly positive impact on the economy, there are growing concerns regarding use of pesticides and their environmental consequences. The conventional methods of pesticide application, like spraying, dusting, and soil injection, cause nontargeted spread of pesticides in the environment.^25^ As a result, less than 0.1% of the applied pesticides reach their target.^26^ It was estimated by Pimentel et al. that one million droplets of insecticides must be applied to target one mosquito.^27^ This off-target deposition added to their extensive use has led to the contamination of aquatic and terrestrial environments, even tracing to drinking water.^28^ Additionally, pesticides are also present in the atmosphere as a result of their high volatility and redistribution by aerial currents and drifts.^29^

Pesticides have shown detrimental effects on the biodiversity of the ecosystem. For example, a study in the Scottish farmlands showed a decline in insect and bird population linked to increasing use of pesticides.^30^ Another study showed decreasing the population of farmland birds in Europe by half.^31^ In the U.S., which accounts for 19% of the estimated global use of the pesticide glyphosate,^32^ there is a significant threat to native species of plants due to its excessive use.^33^ Due to their widespread and excessive usage, trace residues of pesticides can also be found in everyday food items like cooked food, fruit juice, and wine.^34−37^ It has been shown that pesticides can pose significant health risks to humans due to their high toxicity and bioaccumulation in body tissues,^38,39^ resulting in chronic diseases ranging from endocrine disturbances,^40^ teratogenic effects,^40^ neuropathy,^41^ and even some types of cancers.^42^

To solve these problems, several methods have been explored to decrease the environmental presence, including membrane filtration or adsorption (physical), chemical degradation (chemical), and microbial treatment (biological).^43^ Among these, adsorption has proved to be superior in terms of cost and efficacy by providing a noncomplex and more efficient method for pesticide extraction.^44^ For a competent adsorption, several factors must be considered: the adsorbent must be carefully chosen for its high porosity, surface area, the availability of adsorption sites.^45^ In addition, their stability must be considered for agricultural applications to prevent cross-contamination as a result of structural deterioration. Several adsorbents have been investigated for the adsorption of pesticides from the environment, like activated carbon,^46^ zeolites,^47^ and clays; however, there are some drawbacks concerning their applications, as a result of their limited resources, low porosity, stern nonfunctionality, and instability.^48,49^

**Scheme 1:**
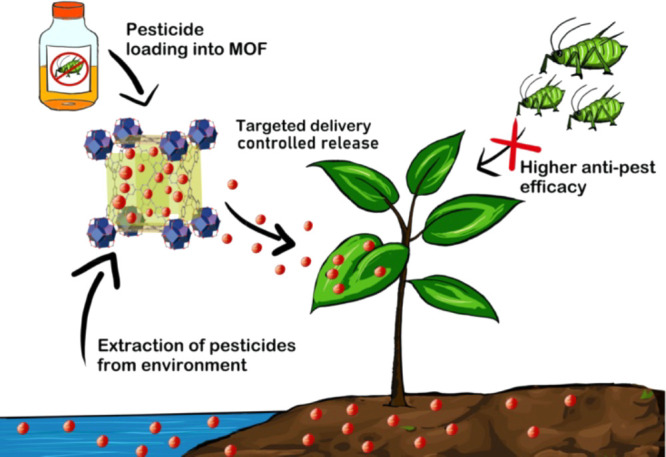
Versatile Nature of MOFs Allows a Wide Range of Agricultural Applications from Extraction of Environmental Pollutant Released from Agricultural Runoff Chemicals to Controlled Release of Pesticides and Other Agrochemicals to Minimize Environmental Impact

In recent years, a relatively new class of materials, metal–organic frameworks (MOFs), have garnered attention due to their high porosity, stability, and fine-tunable nature, making them a favorable candidate for the adsorption and controlled release of pesticides for agricultural applications (Scheme 1).

MOFs are a class of crystalline porous coordination polymers formed by the coordination of multidentate organic linkers with metal cluster nodes, forming a lattice, with void spaces. Due to their scaffold-like structures, MOFs can possess excellent inherent porosities and ultrahigh surface areas, such as the already reported >7000 m^2^ g^–1^ and with a theoretical limit of surface area up to 10,436 m^2^ g^–1^,^50−52^ surpassing long used porous materials like zeolites. In addition, the wide range of customizable selection of metals and organic linkers allows for variable functionality, size, and geometry. The size of pores can be controlled by varying the linker as well as the coordination modes of the metal ions, allowing to develop customizable MOFs suitable for desired applications. For example, UiO-66 and UiO-67, two popular Zr(IV)-dicarboxylate MOFs, were synthesized in 2008 by Lillerud et al.,^53^ and the Langmuir surface area of the latter MOF is increased by approximately 2000 m^2^ g^–1^, just with extension of the linker by one phenyl unit. Subsequently, other UiO-66 and UiO-67 analogues were explored by the functionalization of the linker with several functional groups, such as −NH_2_, −NO_2_, and halides.^54^ Further modification of MOFs can be introduced through postsynthetic modification (PSM) where the presynthesized MOFs can be modified by metal or ligand exchange and/or by other chemical modifications. PSM allows introducing new properties, such as photoactivity, by incorporation of photosensitizer molecules.^55−57^

## 2 MOFs for Pesticide Delivery

Delivery of pesticides using MOFs is a relatively new area of research, and the number of studies continues to increase. MOFs are of particular interest in delivering pesticides and other agrochemicals due to their ultrahigh surface area and tunable pores which can be functionalized to fit specific guest molecules. High surface area and porosity are directly linked to the high loading capacity of MOFs, and the interaction between the MOFs and guest molecules allows slow and controlled release of the agrochemicals. Adsorption and controlled release depend on factors like pore size, surface charge, the p*K*_a_ of the pesticide, and the pH of the media. In addition, the driving mechanisms to achieve maximum loading capacity/extraction capacity are the same, from electrostatic forces, π–π interactions, and hydrogen bonding. Given the highly tailorable nature of MOFs, modifications have been explored to maximize adsorption/extraction ability, using approaches such as linker functionalization,^58^ introduction of linker defects,^59^ development of MOF-derived porous carbons,^60^ and incorporation into composites.^61^ In addition, modifications can give rise to smart multi-stimuli-controlled platforms for controlled release.^62^

Another advantage of using MOFs for delivering agrochemicals is that they have reasonable stability to deliver the agrochemicals, followed by decomposition. This is an advantage, as the decomposition of the MOFs prevents their accumulation in the environment at the end of the application.

Although a new area of research, this application has gained much attention during the past two years, and a number of MOFs have been published recently. A large number of iron-based MOFs have been studied, particularly due to their biocompatibility, followed by zirconium-based MOFs, which show relatively higher stability due to their strong Zr(IV)–O bonds. We have grouped the MOFs for pesticide delivery studies based on the metal ions and provided an overview in the following section, with a summary of key information in Table 1.

**Table 1:** List of MOFs Used for Delivery of Pesticides

MOF used	Metal	BET surface area (m^2^ g^–1^)	composite	agrochemical loaded	loading capacity (wt %)	efficacy	stimuli triggers	ref/year
OPA-MOF	Fe			P and N fertilizers	P: 12.5	plant growth		(65)/2015
					N: 3.1	soil enrichment		
					oxalate: 14.5			
MIL-100	Fe	2251^a^		azoxystrobin	16.2	antifungal activity against *F. graminearum* and *P. infestans*	pH responsive	(66)/2020
		1199^b^				plant growth		
NH_2_-MIL-101	Fe	953.9^a^	PDA	diniconazole	14.7	good antifungal activity against *Fusarium graminearum*	pH responsive	(67)/2020
		449.8^b^						
		13.0^c^						
MIL-101	Fe	800^a^	Fe^3+^-TA	tebuconazole	24.1	high efficacy against *Rhizoctonia solani*, *Fusarium graminearum*, and control of *Blumeria graminis*	pH	(69)/2021
		70^b^					sunlight (NIR)	
		30^c^					H_2_O_2_	
							GSH	
							phosphates	
							EDTA	
MIL-101	Fe	417.42^a^: unloaded composite	CMCS	dinotefuran	24.5	effective against late stage pest-outbreak	pH	(68)/2020
		55.60^b^: loaded composite				protection against photodegradation of dinotefuran	citric acid	
Fe-MOF	Fe	93.31^a^		tebuconazole	29.7	reducing tebuconazole toxicity effects on plant growth		(71)/2022
		47.99^b^						
MIL-101	Fe	2383.10^a^	silica	chlorantraniliprole	23	higher efficacy against *P. xylostella* larvae than applied pesticide	pH	(70)/2021
		731.82^b^				protection against photodegradation		
PCN-224	Zr	1533.62^a^	pectin and chitosan composite	tebuconazole	30	double microbicide activity	pH responsive	(77)/2019
		106.55^b^					pectinase	
		65.17^c^					light	
UiO-66	Zr	926.54^a^		λ-cyhalothrin	87.1	effective against *Musca domestica* and Aphis craccivora Koch		(78)/2020
		526.57^b^				long-term insecticidal effect compared to commercial pesticide formulation		
UiO-66-NH2	Zr	824.35^a^	lignosulfonate (SL)	thiamethoxam	30.57 (MOF) and 33.56 (composite)	42 day no-pest against rice planthopper	pH responsive	(79)/2021
		401.43^b^						
		235.59^c^						
UiO-66	Zr	502.51^a^	Fe_2_O_3_	imidacloprid	15.87	comparable LC_50_ to commercial imidacloprid		(80)/2021
		264.01^b^	polydopamine (PDA)			magnetic composite easily collectable		
UiO-66	Zr	502.5^a^	Fe_2_O_3_	imidacloprid	15.87	comparable LC_50_ to commercial imidacloprid		(80)/2021
		264.01^b^	polydopamine (PDA)			magnetic composite easily collectable		
UiO-66-NH2	Zr	865^a^	PCL (postsynthetic loading)	MCPA	release at 72 h in water: 0.056 mg mL^–1^	PCL enhanced release in water due to swelling		(81)/2022
		619^b^						
UiO-66	Zr	1456^a^	PCL (postsynthetic loading)	MCPA	release at 72 h in water: 0.043 mg mL^–1^	PCL enhanced release in water due to swelling		(81)/2022
		1100^b^						
MOF-1203	Ca	160		1,3-DCPP	13			(82)/2022
MOF-1201								
HKUST-1	Cu		TMPyP		12	photosensitizer pesticide effect	light	(83)/2021
						good efficacy against *Sclerotinia sclerotiorum*		
NH_2_-Mil-101(Al)	Al	2359.0^a^		azoxystrobin diniconazole	29.72	EC_50_: 0.065 mg·mL^–1^ effective against *Rhizoctonia solani*	pH	(84)/2021
		468.7^b^			6.71			
MOF-5	Zn	121.28^a^	PVA/ST	atrazine	60.12			(88)/2022
		106.32^b^						
ZIF-8	Zn	1775^a^	PMMA/zein	dinotefuran	16.19	protection of pesticide against UV degradation	pH	(85)/2022
		645^b^		Zn^2+^	21.13	32 day antipest effect against	protease	
		128^c^						
ZIF-67	Co			boscalid	18	antifungal effect 4–6 times higher than commercial product	pH	(86)/2022
CuBTC	Cu			avermectin	40	protection against photodegradation (retention 69.4%, pH 9.0, 120 h)	pH-dependent	(87)/2022
						-anti pest effect against *B. xylophilus* for 12 days		
GR-MOF-7	Cu			glufosinate		40 and 24% inhibition of *Staphylococcus aureus* and *Escherichia coli* at ≤2.5 ppm		(89)/2022
						inhibit germination of *R. sativus*		
Ni-ITQ-HB	Ni	285^a^		3-(*S*)-methyl-6-(*R*,*S*)-isopropenyl-9-decenyl acetate	25			(101)/2016
IRMOF-NHPr		1914^b^		3-octanone	62	mph loaded MOFs showed similar results to conventional mph applications, drawing similar number of insects to bait		(102)/2020
IRMOF-3		2613^b^		4-methyl-3-heptanone (mph)	75			

a BET surface area before loading.

b BET after loading.

c BET surface area after integration of MOFs into the composite.

### 2.1 Iron-Based MOFs

**Figure 1:**
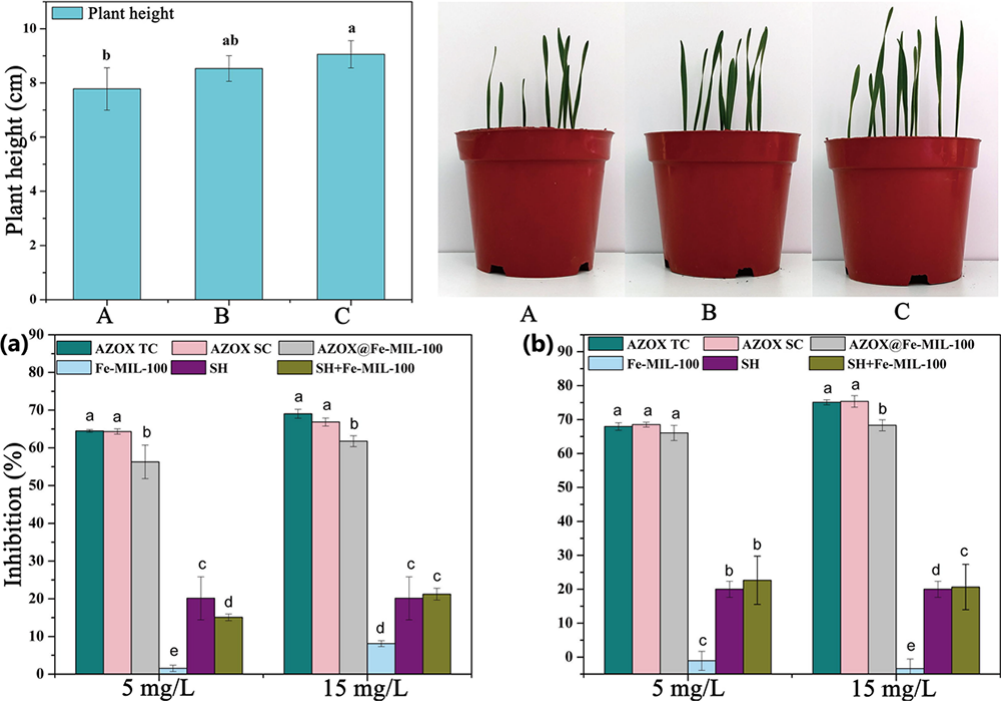
Top: Effect of plant height as a result of treatment with Fe-MIL-100. Bottom: Measurements of antifungal activity of AZOX@Fe-MIL-100 and other formulations, AZOX SC and AZOX TC against (a) *P. infestans* and (b) *F. graminearum* on the fourth and seventh day, respectively. Reproduced (including photo) and adapted with permission from ref (66). Copyright 2020 Elsevier.

Iron-based MOFs are particularly interesting for their biocompatibility,^63^ and as they can act as a good slow-releasing source of iron, a micronutrient, critical for plant growth and survival.^64^ One of the earliest studies in 2015 by Anstoetz et al. demonstrated the use of an iron-based MOF (OPA-MOF) for potential agrochemical delivery.^65^ Made up of an iron-phosphate core linked by oxalate, OPA-MOFs demonstrated the slow release of N (urea) and P (phosphate) fertilizers by microbial degradation of oxalate. It was shown that the release of N was rapid; however, the bioavailability of P was much less than conventional phosphate fertilizers, which might be attributed to the acidification of soil by the degradation of the MOF. Nonetheless, it was a successful demonstration of the potential of MOFs for slow-release agricultural applications. In 2020, Shan et al. reported the loading of a high surface area Fe-MIL-100 (BET surface area of 2251 m^2^ g^–1^) with the fungicide azoxystrobin.^66^ The loading was performed by magnetically stirring Fe-MIL-100 in a solution of azoxystrobin. Determination of loading content was done by ultrasonically dispersing 5 mg of loaded Fe-MIL-100 in 5 mL of methanol for 1 h. The supernatant was collected, and the process was repeated four times. The cumulative supernatant concentrations were combined and analyzed with HPLC, where it was found that the highest loading was 16.2% by weight using a 3:1 ratio of pesticide to MOF. Extended release studies showed initial pH-dependent burst effects, followed by a sustained release of the fungicide. Bioactivity studies show similar antifungal activity to available azoxytrobin formulations against *P. infestans* and *F. graminearum*. Fe-MIL-100 also demonstrated its ability as a source of iron micronutrient, which resulted in a 16.4% increase in plant height (Figure 1).

In a later study by Shan et al.,^67^ controlled release properties were enhanced by the elimination of burst effects, by integrating diniconazole-loaded NH_2_-MIL-101 with polydopamine (PDA). Loading of diniconazole into the amine-functionalized NH_2_-MIL-101 was performed at a ratio of 1:1 in dichloromethane at room temperature, achieving a loading of 28% by weight. As observed in the previous study, loading content increased with higher pesticide to MOF ratios. This was postulated to be due to the higher concentration of diniconazole promoting greater adsorption into MOF pores. Incorporation of PDA resulted in a decrease of the diniconazole loading to less than 15%. However, PDA functionalization proved to decrease the initial burst release effect when compared to the unmodified loaded NH_2_-MIL-101. In addition, these systems also exhibited a pH-responsive release rate, with the highest release rates achieved in acidic media. MIL-101 was also utilized by Feng et al.^68^ to be loaded with the pesticide dinotefuran (DNF) and encapsulated within carboxymethyl chitosan (CMCS). After the solvothermal synthesis of MIL-101 from terephthalic acid linker and iron(III) chloride precursors, loading was performed by stirring 0.1 g of MOF into 30 mL of 25 mg mL^–1^ DNF solution in ethanol overnight. Encapsulation was then carried out by dropwise addition of 25 mL of CMSC solution. This method yielded individually coated MOF granules, with DNF-loaded CMCS.

**Figure 2:**
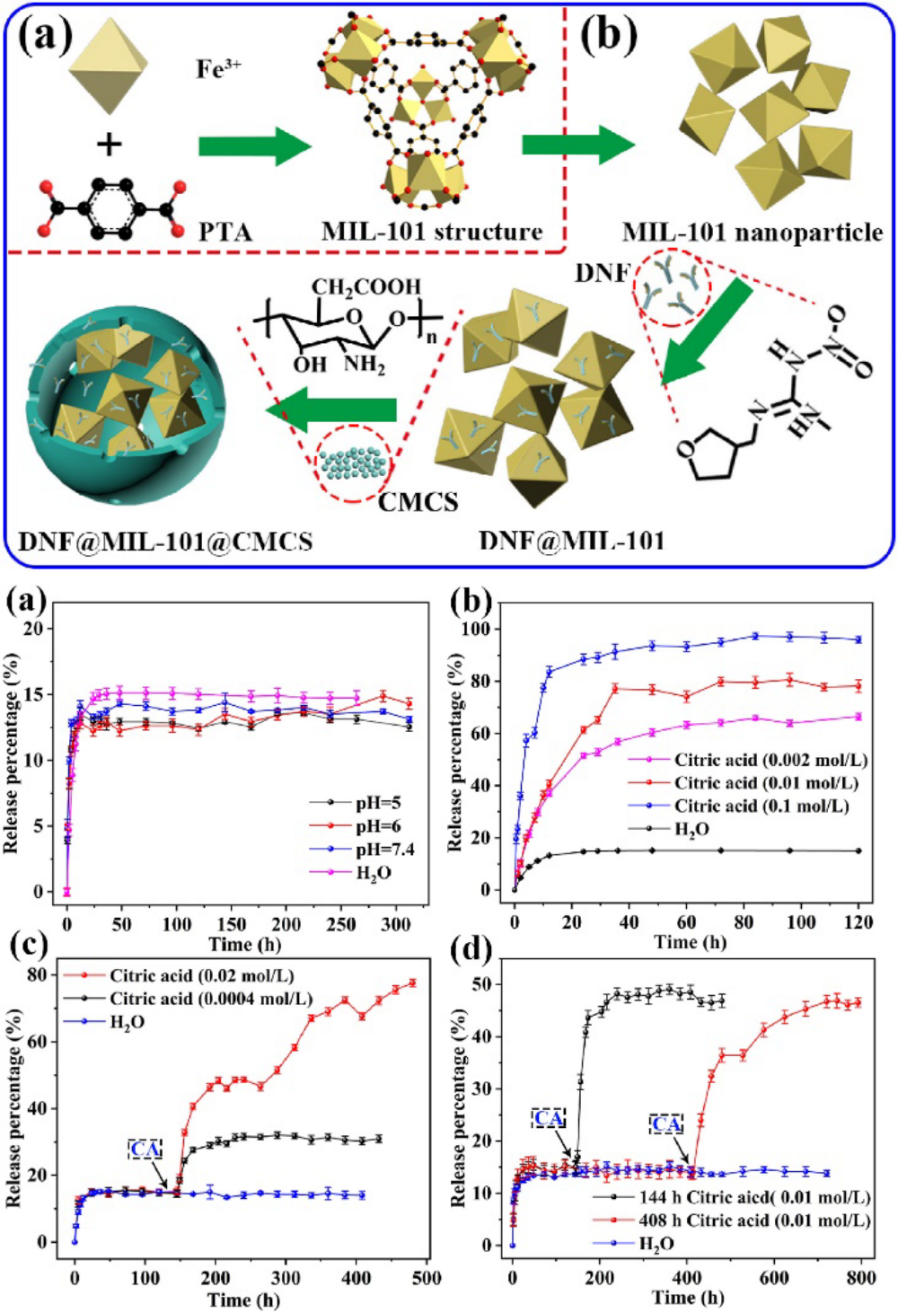
Top: Scheme showing the synthesis step of DNF-loaded CMCS@NH_2_-MIL-101. Bottom: (a) Effect of pH on the release of DNF over time, (b) effect of different concentrations of citric acid on release percentage, (c) stimulated DNF release by different concentrations of citric acid at the same time, (d) release of DNF overtime, stimulated by the intermittent addition of citric acid of the same. Reproduced and adapted with permission from ref (68). Copyright 2020 Elsevier.

The loading capacity was measured to be 24.5%. The release studies showed pH-sensitive slow release of DNF, as presented in Figure 2. The release rate was found to be stimulated by the addition of citric acid, resulting in enhanced degradation of the CMCS coating. Interestingly, the photostability studies done on contained DNF showed that it was protected against UV degradation, proving that the encapsulation inside the micropores enhances its agricultural impact and preserves the pesticide in environmental conditions.

Release of DNF from the composite consisted of two stages: an initial step of ∼14% release from the CMCS coating and a slower, more controlled release in the second stage stimulated by citric acid, with ∼83% DNF released from inside the MOF pores. Finally, the MOF composites showed good antipest activity, especially for a late-stage pest outbreak. Efficacy studies on rice-planthopper-infected plants show growth over a period of 41 days, owing to their insecticidal effect. The insecticidal effect of CMCS@NH_2_-MIL-101 did not deplete with time as much as conventionally applied DNF, which is due to the stimulated release maintained upon the addition of citric acid.

**Figure 3:**
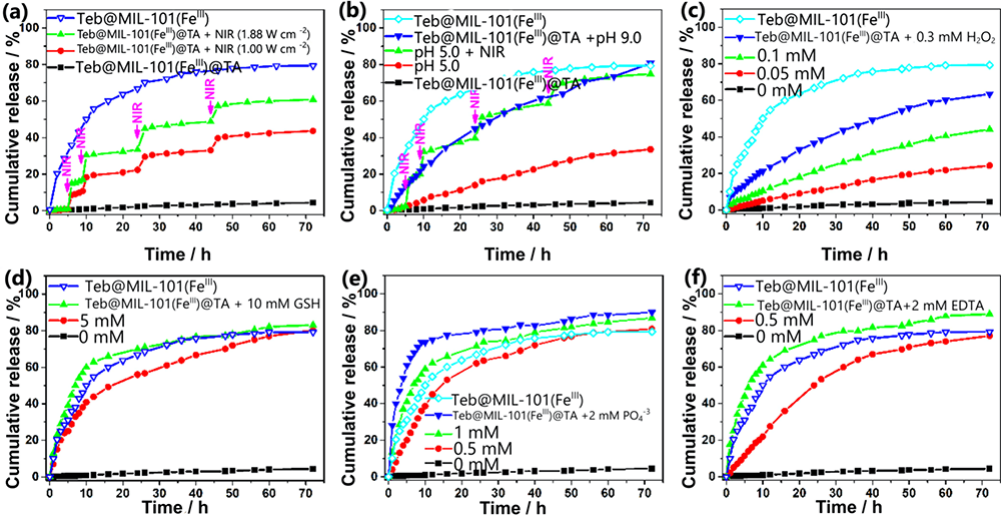
Stimulated release of tebuconazole over time, investigating the effect of (a) NIR irradiation, (b) pH, (c) presence of H_2_O_2_, (d) glutathione (GSH), (e) phosphate, and (f) EDTA, as well as the effect of NIR on release. Reproduced and adapted from ref (69). Copyright 2021 American Chemical Society.

In 2021, Dong et al. developed gated ferric tannic acid (TA) smart networks, incorporated into a defective MIL-101 lattice.^69^ MIL-101 was loaded with the fungicide tebuconazole, followed by the formation of metal-phenolic networks by the capping of uncoordinated metal sites with Fe^3+^-TA. The capping helped to prevent premature release of fungicide, acting as a gate for slow release of tebuconazole, as well as multi-stimuli-responsive gates for controlled release of fungicide (Figure 3).

The fungicide release was enhanced by destruction of metal-phenolic networks. Changes in pH can cause Fe^3+^-TA disassembly due to transition to mono-, bis-, and tricomplex states, thus weakening the metal-phenolic networks and allowing for more tebuconazole release. Other factors like the presence of H_2_O_2_, GSH (glutathione), phosphates, and EDTA affect the release of pesticide by affecting the integrity of the metal-phenolic networks as well as MIL-101, resulting in variable multi-stimulus-controlled release conditions. The pH ranging from 5 to 9 enhanced the release. In addition, oxidative stress in plants causes the production of H_2_O_2_ and GSH as defensive mechanisms, and as a consequence of the Fenton reaction, this further enhances the degradation of the MOFs and the release of the pesticide. Similar to the previous study, photostability testing showed that the incorporation of pesticide inside the pores protected the pesticide from photodegradation, as Fe^3+^-TA was able to block UV radiation. Further studies using near-infrared (NIR) radiation show that the NIR radiation enhanced the release of tebuconazole upon intermittent illumination (see Figure 3). This was due to degradation of the metal-phenolic networks caused by thermal diffusion from NIR. This photothermal effect was directly proportional to the power density of the laser used. Antifungal activity testing showed an excellent efficacy against *R. solani* and *F. gaminearum*, with an ED_50_ of 0.4960 and 0.5658 mg L^–1^ after 48 h, respectively. This study is a good example for the potential of adapting MOFs for environmental conditions and applications.

Silica-coated MIL-101 composites, MIL-101@silica, were used for the delivery of chlorantraniliprole.^70^ The loading capacity was shown to be 23%, as a result of the high porosity of silica and MIL-101. Release studies show a higher release in alkaline conditions, as a result of the structural degradation of the silica and the decomposition of the iron-carboxylate moiety of the MOF structure. UV photostability studies show that the encapsulation of the pesticide helped protect it against degradation, showing a decomposition rate of <24% in 20 min, compared to that of bare chlorantraniliprole, which showed complete decomposition. Compared to suspensions of chlorantraniliprole, the loaded MOF composites showed a higher mortality rate (86%) compared to ∼37% against *P. xylostella*. In addition, the MOF composites were shown to be safe for use on crops.

In 2022, Zhao et al. reported an iron-based MOF loaded with the fungicide tebuconazole.^71^ The loading content of the MOF was ∼30% by weight, with controlled release studies showing slow sustained release, reaching ∼91% in 30 h. The fungicidal effect of loaded MOFs was tested on wheat seedlings. When compared to conventionally applied tebuconazole, the loaded MOFs reduced the phytotoxic impact of tebuconazole on the seeds. In addition to the antifungal effect of the loaded MOF, the plants showed an enhanced growth in length, weight, and chlorophyll content, as a result of the iron supplementation that the MOFs provided.

### 2.2 Zirconium-Based MOFs

Zr-based MOFs have garnered attention in recent years, owing to their high thermal and chemical stability^72−75^ Through their strong covalent bond between the Zr(IV) cation cluster and the dicarboxylate ligands,^76^ Zr-based MOFs are a unique class of robust polynuclear porous crystals with a wide range of potential applications.^75^

One of the earliest studies using Zr-based MOFs for agrochemical release was reported in 2019.^77^ Tang et al. modified the porphyrinic MOF, PCN-224, for stimuli-responsive controlled release of tebuconazole fungicide. PCN-224 was synthesized from zirconyl chloride octahydrate and *meso*-tetra(4-carboxyphenyl)porphyrin (H_2_TCPP) linkers, modulated by benzoic acid. Loading of fungicide was performed by stirring PCN-224 in ethanol solution of tebuconazole, yielding a 30% loading capacity. The composite was prepared by layer-by layer assembly of pectin and chitosan. The dual antimicrobial effect of the composites was observed to be enhanced by two routes: (i) the slow release of tebuconazole, resulting from pectin digestion due to microbial pectinases enhancing the release, and (ii) the photodynamic activation of the porphyrin linker, resulting in the production of (^1^O_2_) reactive singlet oxygen when exposed to light. Changes in pH and the presence of light seemed to have an effect on the pesticide release. At 174 h, release in PBS solution (pH of 7.0) was only ∼3% in the absence of pectinase, compared to >17% at pH of 5.0. When pectinase was added to PBS solution of 5.0 pH, the release was 87% at 174 h, reaching equilibrium. Antimicrobial studies demonstrate both antibacterial and antifungal properties, with the fungicide-loaded composites exhibiting an efficacy of 57 and 25% in light and dark, respectively, against *X. campestris pv campestris* bacteria, while exhibiting antifungal efficacy of 68 and 51% in light and dark, respectively, for *A. alternate*. Experiments done on the safety of the composite on crops on Chinese cabbage proved its potential to be used, with no potential damage to the plants.

In 2020, Meng et al. reported loading of the insecticide λ-cyhalothrin (LC) in UiO-66.^78^ UiO-66 was synthesized using ZrCl_4_ and terephthalic acid as organic linker. Pesticide loading was optimized, achieving a high loading content of 88%, using a pesticide to MOF ratio of 30:1 in DMF while stirring for 24 h. Due to the limited solubility of LC, release studies were done in 60% DMF aqueous solution. Equilibrium release of 70% was achieved after 72 h. The insecticide efficacy was tested against *Musca domestica* (housefly) and *Aphis craccivora* Koch (agriculture pests), showing better antipest effect than conventional formulations with an LC_50_ value (lethal concentration that kills 50% of the animals) equivalent to free LC at 72 h. In addition, the insecticidal activity was enhanced with time, as a result of the sustained release.

**Figure 4:**
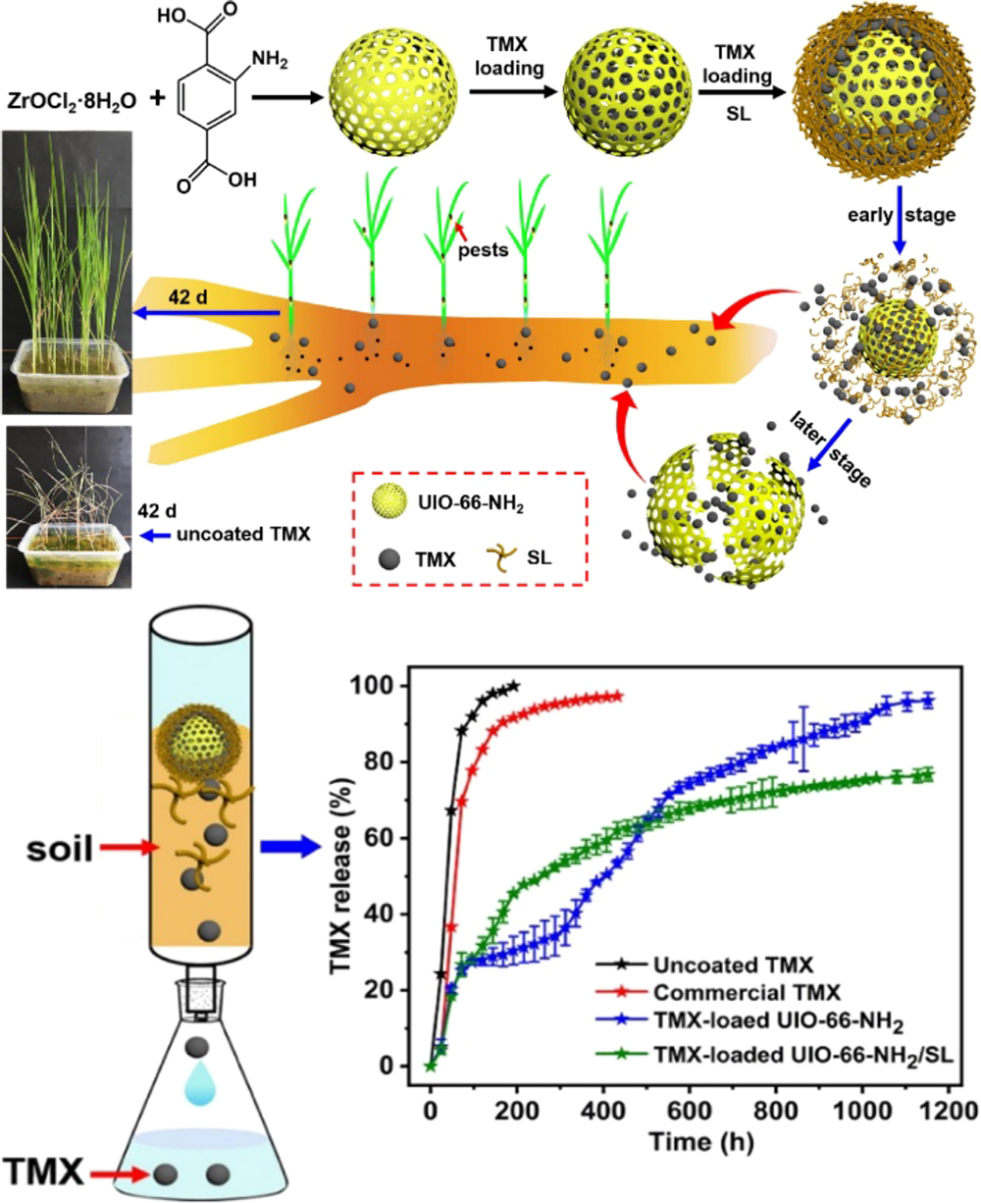
Top: Scheme showing the synthesis and degradation mechanisms of TMX-loaded UiO-66-NH_2_/SL. Bottom: Time release studies. Reproduced (including photo) and adapted with permission from ref (79). Copyright 2021 Elsevier.

Amine-functionalized UiO-66-NH_2_ was used for the controlled release of the pesticide thiamethoxam (TMX).^79^ UiO-66-NH_2_ was synthesized from ZrCl_4_ and 2-aminoterephthalic acid using solvothermal synthesis. Loading of TMX was performed by stirring 1.5 g of MOF in a 300 mg mL^–1^ solution of the pesticide in methanol (Figure 4). Functionalization of the loaded MOFs was carried out by stirring in solution of TMX and acetic acid in methanol, with the dropwise addition of 50 mL of sodium lignosulfonate (SL) at a concentration of 10 mg mL^–1^. The yielded microcapsules were double layered as a result of the cross-linking of the sulfonate group of the lignosulfonate with the protonated UiO-66-NH_2_. This functionalization helped to create a double layer for slow, long-term controlled release of TMX. Experiments investigating the release in soil showed the cumulative release at 1152 h was 96% for uncoated TMX-loaded UiO-66-NH_2_, while for TMX-loaded-UiO-66-NH_2_/SL, it amounted to less than 77%. This proved that the slow release of pesticide can be extended as a result of the double layer coating, as well as the rate of degradation of those layers. Oddly, it was observed that TMX-loaded UiO-66-NH_2_/SL had a release rate higher than that of the uncoated counterpart in the first 600 h, perhaps as a result of microbial degradation of the double layer. This lower initial rate resulted in 42 day antipest activity, in addition to an increase of 41 cm in plant height. In contrast, uncoated loaded UiO-66-NH_2_ showed an antipest effect of only 16 days, with only 21 cm growth in plant height.

In 2021, Meng et al. reported the use of UiO-66 for incorporation into nanocomposites by coating with Fe_2_O_3_ and PDA (polydopamine).^80^ The composites were loaded with the pesticide imidacloprid (IMI) by dispersing 10 mg of MOF composite in 10 mL of IMI/DMF solution at a concentration of 1 mg L^–1^ and stirring for 24 h at room temperature. The resultant loading capacity was ∼16%. The release activity was studied using dialysis in water. At 48 h, the cumulative release was about 50% for IMI@Fe_2_O_3_@PDA@UiO-66 compared to 80% for free unloaded IMI. Anti-insect efficacy was compared against *Aphis craccivora* Koch, with IMI@Fe_2_O_3_@PDA@UiO-66 showing an LC_50_ of 2.15 mg/L compared to that of commercial IMI water-dispersible granules (2.19 mg/L). Functionalization with iron nanoparticles also allowed for the composite to be retrieved, with 95% retrieval rate with the help of a D25 magnet, thus proving the potential of being a cleaner alternative to conventional pesticide application methods.

**Figure 5:**
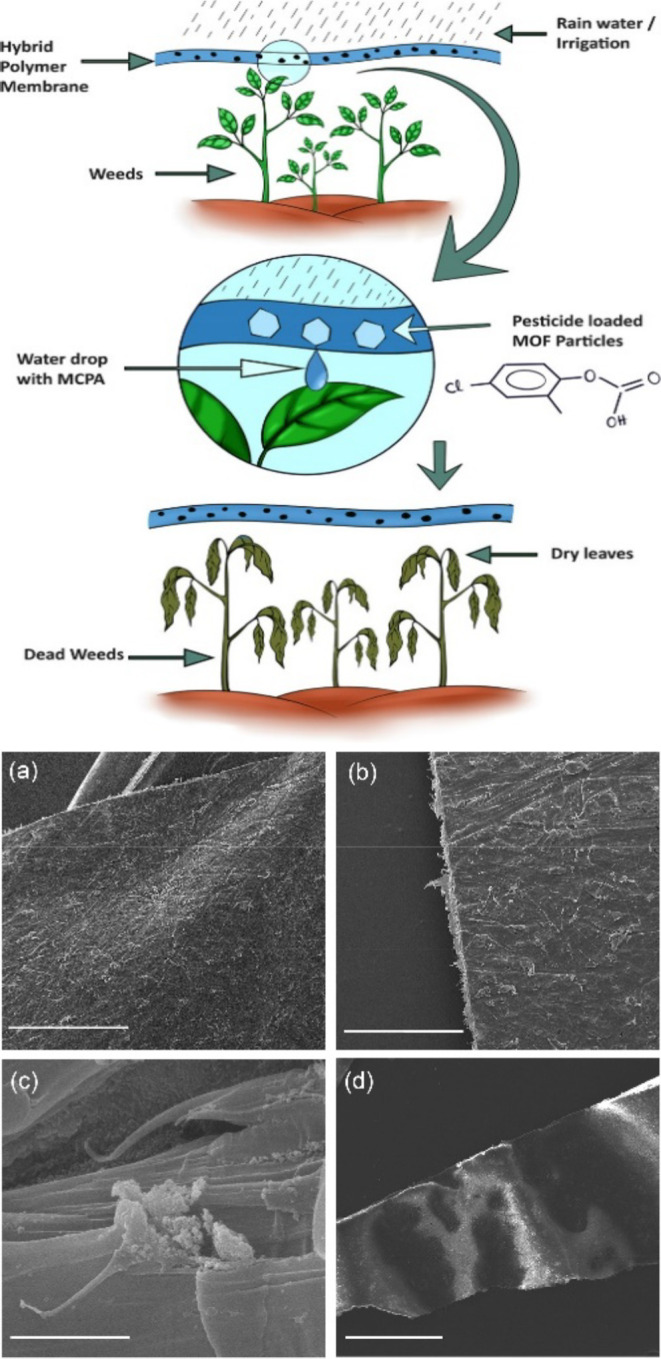
Top: Scheme showing the use of MOF composites, for the controlled release of herbicide, MCPA. Bottom: SEM images of polycaprolactone, MCPA-loaded MOF composites. Scale bars: (a, d) 1 mm, (b) 500 μm, and (c) 5 μm. Reproduced and adapted from ref (81). Copyright 2022 American Chemical Society.

Finally, in 2022, three Zr-Based MOFs, UiO-66, UiO-66-NH_2_, and UiO-67, were loaded with the herbicide 2,4-methylchlorophenoxy acetic acid (MCPA).^81^ Two methods were employed for the loading of MCPA: postsynthetically, by stirring in a solution of MCPA in ethanol, and in situ, where the modulator is replaced by MCPA. The MOFs were then incorporated into biodegradable polymer sheets of polycaprolactone (PCL), and the release was tested in water and ethanol over 72 h (Figure 5). The postsynthetically loaded UiO-66-NH_2_ showed the best release with concentrations of 0.056 and 0.037 mg mL^–1^ in ethanol and water, respectively. When incorporating the MOFs into PCL, the release in water was found to be enhanced compared to that in ethanol; this was attributed to the swelling behavior of PCL in water.

### 2.3 Other Miscellaneous Metals

Apart from iron- and zirconium-based MOFs, a number of other MOFs have been reported for loading and delivery of agrochemicals. One of the earliest studies by Yang et al. in 2017 reported the use of two MOFs, MOF-1201 and MOF-1203, assembled from calcium ions, and l-lactate, with acetate bridging between the clusters.^82^ These two MOFs exhibit BET surface areas of 430 and 160 m^2^ g^−1^, respectively. Loading of the fumigant, *cis*-1,3-dichloropropene, was performed via measuring the *cis*-1,3-dichloropropene sorption isotherms at room temperature. The isotherms showed an uptake of 13% by weight at *P*/*P*_0_ = 0.1, attributed to adsorption within the pores. Slow release studies were performed by purging the loaded MOFs in an air flow of 1.0 cm^3^ min^–1^ and observing the weight loss by thermogravimetric analysis. When compared to a liquid *cis*-1,3-dichloropropene, MOF-1201 showed a 100 times slower release rate. For example, 80% of total weight of MOF-loaded *cis*-1,3-dichloropropene was evaporated in 100,000 min, compared to 1000 min for the liquid *cis*-1,3-dichloropropene. The degradability of MOF-1201 was tested in water, showing its ability to disassemble into its environmentally friendly building blocks, calcium ions, acetate, and lactate. These two MOFs demonstrated their ability for slow release of pesticide and, as a precursor to supply calcium, as a macronutrient for plant growth.

In 2021, nanocomposites of the zinc-based MOF, Zn-HKUST-1, were synthesized,^83^ in which the porphyrin 5,10,15,20-tetrakis(1-methyl-4-pyridinio)porphyrintetra(ptoluenesulfonate) (TMPyP) is incorporated within the cage structure of the MOF. Synthesis was done by in situ incorporation of TMPyP with zinc acetate dehydrate and benzene dicarboxylic acid linkers. TMPyP acts as a photosensitizer, with the ability to produce a singlet oxygen upon light activation and thus exhibiting antimicrobial action. TMPyP loading was 12% by weight, with studies showing excellent dose-dependent antibacterial and antifungal activity against *S. sclerotiorum*, *P. aphanidermatum* and *B. cinerea* and *P. syringae pv lachrymans* and *C. michiganense* subsp. *michiganese* upon photodynamic activation. Chromosome assays proved no genotoxicity on crops of cucumber and Chinese cabbage, proving to be safe to use on plant crops.

MIL-101(Al), an aluminum-based MOF, with a 2-aminoterephthalic acid linker and a BET surface area 2359.0 m^2^ g^–1^, was loaded with two fungicides, diniconazole and azoxystrobin,^84^ by centrifuging 30 mg of azoxystrobin, diniconazole, and MIL-101(Al) in 1 mL of dichloromethane for 6 h. Loading content was ∼7 and 30 wt % for azoxystrobin and diniconazole, respectively. The BET surface area after loading decreased to 469 m^2^ g^–1^. Azoxystrobin and diniconazole mixtures were used due to their higher effective combined effect. AZOX@Dini@NH2-Al-MIL-101 showed an EC_50_ lower than that of the fungicide mixture. Antimicrobial studies showed that empty unloaded MOFs exhibit fungicidal activity against *R. solani*, suggesting that the lower EC_50_ value might be a result of the MOF itself contributing to antifungal activity.

The zeolitic imidazolate framework ZIF-8 was used for the slow and controlled release of dinotefuran (DNF) by encapsulating the MOF in a poly(methyl methacrylate) (PMMA) shell.^85^ The composites were also coated with a hydrophobic film called zein, which is a biodegradable byproduct of corn and can be only digested by the proteases present in the guts of pests, allowing for a more targeted approach for pesticide delivery. The loading capacity of DNF was measured to be 16% by weight, with the composites possessing excellent protease and pH-triggered release, exhibiting prolonged pesticide release over a period of 32 days when tested in soil. DNF inside the composites was protected against photodegradation, with photostability studies showing a retention percentage of 78% of DNF when irradiated for 48 h, compared to only 8% for free DNF. The composites also showed good leaching resistance compared to free DNF formulations. The composites proved to be safe for use in crops, and furthermore, they proved to be a good supply of Zn^+^ micronutrient for enhancing the crop growth.

Another ZIF, ZIF-67, was used by Zhang et al. for the loading of the fungicide boscalid.^86^ Under optimum conditions for loading, the loading content was 18% by weight. The effects of pH change were tested, and the cumulative release rate was shown to be between 25 and 38% for pH ranges of 7–3.5, with antifungal studies showing that this method of controlled delivery was 4–6 times more effective than the commercially available formulation of boscalid. The MOFs were tested against *Botrytis cinerea* (gray mold). During its propagation, the fungus releases oxalic acid, thus lowering the pH. This facilitated the disintegration of the framework and resulted in heightened fungicide release, allowing for a more controlled site-specific route of fungicide delivery.

**Figure 6:**
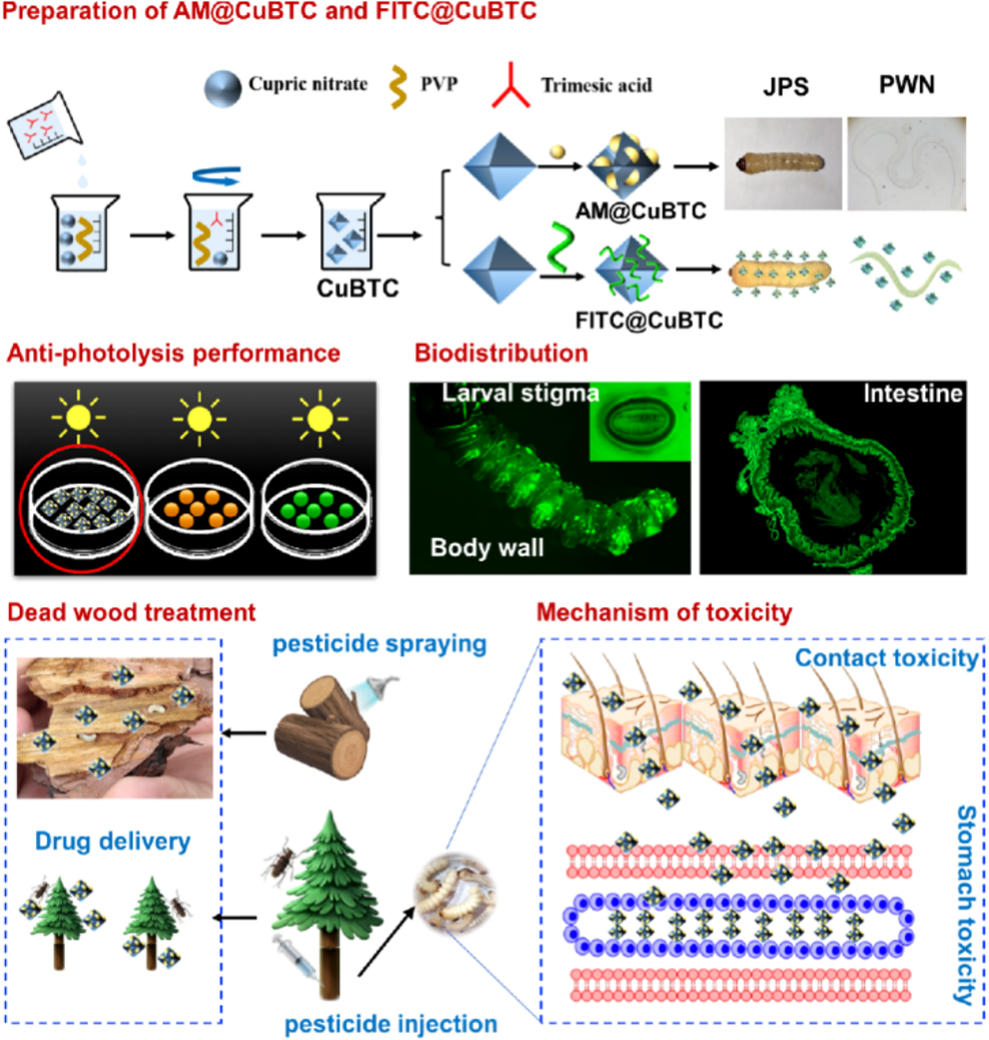
Scheme summarizing the studies done on the avermectin-loaded CuBTC, as well as their potential for targeted controlled delivery. Reproduced (including photo) and adapted with permission from ref (87). Copyright 2022 Creative Commons Attribution License.

Liu et al. loaded the MOF CuBTC with avermectin against the insect *Bursaphelenchus xylophilus*.^87^ The controlled release was found to be pH-dependent, with a cumulative release of 92% in 12 h. In addition, the loading of avermectin inside the MOFs protected it against photodegradation, with a retention of 69% in 120 h at a pH of 9.0. This study explored the potential of targeted controlled delivery to insect larvae intestines by injecting infested and dead wood with the loaded MOFs, as shown in Figure 6.

In a study reported by Lee et al.,^88^ MOF-5, a zinc terephthalate MOF, was loaded with the herbicide atrazine by dispersing 0.2 g of MOF-5 in 30 mL of 0.030 g/L of atrazine/methanol solution for 3 h. The loaded MOF-5 was then incorporated into a poly(vinyl alcohol)/starch (PVA/ST) composite through electrospinning. Herbicide release studies have shown that incorporating the herbicide inside the MOFs and then into composites provided release rates lower than those from mixing atrazine into the polymer matrix.

In 2022, Sierra-Serrano et al. integrated the herbicide glufosinate as a building block for a copper-based two-dimentional MOF, named GR-MOF-7,^89^ by incorporating glufosinate as a ligand in the MOF structure, ensuring high loading capacity and providing a greener one-pot synthesis method, while using the metal cation and the linker to produce a combined synergistic effect. GR-MOF-7 proved to be water stable for up to 5 days. Antibacterial properties against *Staphylococcus aureus* and *Escherichia coli* showed a growth inhibition of 40 and 24%, respectively. In addition, its herbicide activity on *Raphanus sativus* weed was also studied, showing the combined inhibitory effect of the Cu^2+^ cation and the herbicide ligand, inhibiting 100% of seed germination at a concentration of 0.1 M, which is a lot higher than that of free glufosinate, resulting in 32% inhibition.

The studies described above have shown the potential of MOFs as a vehicle for slow release of agrochemicals by incorporating the loaded MOFs into different formulations, composites, and matrices to create multistimulus slow-release platforms which are adaptable to the agricultural surrounding. Factors such as pH, microbial presence, and light can potentially influence the release of the loaded pesticides, and modification using different polymers and materials can also be used for further control and enhancement of release properties.

### 2.4 MOFs for Release of Antipest Semiochemicals

Semiochemicals are a class of organic compounds that act as chemical messages for communication within or between different species of organisms.^90^ They range from aldehydes, alcohols, and terpenes to complex structures like proteins.^91^ Semiochemicals are divided into two categories: pheromones, which mediate interactions between individuals of the same species, and allelochemicals, which signal communications between different species. Sex pheromones, a subset of pheromones, have been studied widely for their potential as a cleaner, more environmentally friendly pesticide.^92^ Sex pheromones are produced by female insects to attract males for courtship.^93^ They are diffused via air, forming strands of odor that stretch and spread, dispersed between pockets of clean air.^94^ The most common and efficient method of using pheromones for pest control is mass trapping,^95^ where insects are lured into physical traps that capture them, decreasing their population density and hence reducing the damage caused by pests.^96^ Several cases in the literature have utilized porous nanostructures, such as silica and zeolites for the slow release of semiochemicals.^97−100^ However, there are only a very few studies reported employing MOFs to date. In 2016, Moreno et al. synthesized 1D nickel-based metal–organic nanoribbons,^101^ resulting in an inorganic metal cluster chain attached to organic spacers made of a monocarboxylic acid. Synthesis resulted in ordered layers that can expand and exfoliate when treated with different solvents. The pheromone 3-(*S*)-methyl-6-(*R*,*S*)-isopropenyl-9-decenyl acetate was loaded into 2 g of material at a loading content of 25% by weight. Release kinetics showed a release retention lower than that of other mesoporous materials, with retaining only 10% of the loaded pheromone, hence proving high potential for slow and controlled release of chemicals for pest control applications using this new class of materials.

**Figure 7:**
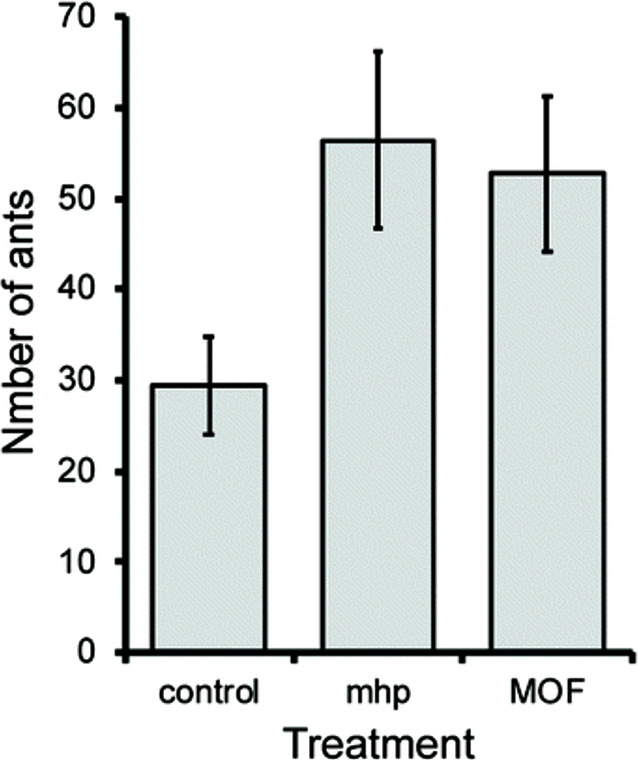
Comparison of the antipest activity of mhp-loaded MOFs and conventional mhp application treatments in attracting insects to bait. Reproduced and adapted with permission from ref (102). Copyright 2020 Creative Commons Attribution License.

In another study,^102^ zinc-based MOFs, including IRMOF-3 and IRMOF-NHPr, as well as zirconium-based MOFs (UiO-66 and UiO-66-NHPr) were used for loading the pheromones, 3-octanone and 4-methyl-3-heptanone. Optimization of the loading process was performed, and it was determined that the best uptake was achieved by using 4:1 pheromone to MOF ratio in 5 mL of DMF when left still for 3 days. To find the loading content, the zinc-based MOFs were digested with DCl and analyzed by ^1^H NMR. Loading content was measured to be 75% and 62% (w/w) for IRMOF-3 and IRMOF-NHPr, respectively. The zirconium-based MOFs were digested in ammonium fluoride due to their high stability in acid. The results showed a loading of 35.1% for UiO-66-NHPr. Biological activity studies were performed on the 4-methyl-3-heptanone (mhp)-loaded MOFs. It was shown that the loaded MOFs were successful in attracting insects to the bait, with similar results as pure mhp, as shown in Figure 7.

To date, there have not been many studies investigating using MOFs as nanocontainers for the slow release of pheromones. However, the catalytic properties of MOFs were employed in the catalysis of pheromones. In a study,^103^ the iron based MOF, MIL-101(Fe) was used along with a polyoxometalate (POM) cocatalyst, for the highly site-selective catalytic conversion of 3*Z*,6*Z*,9*Z*-octadecatriene into 6,7-epoxide. Both chemicals are prime sex pheromones of *E. obliqua Prout*, and their mixtures have been used for their efficiency in mass trappings. Efficacy studies have showed a considerable enhancement of insect attraction compared to commercially sold mixtures.

## 3 MOFs for Extraction of Pesticides

**Figure 8:**
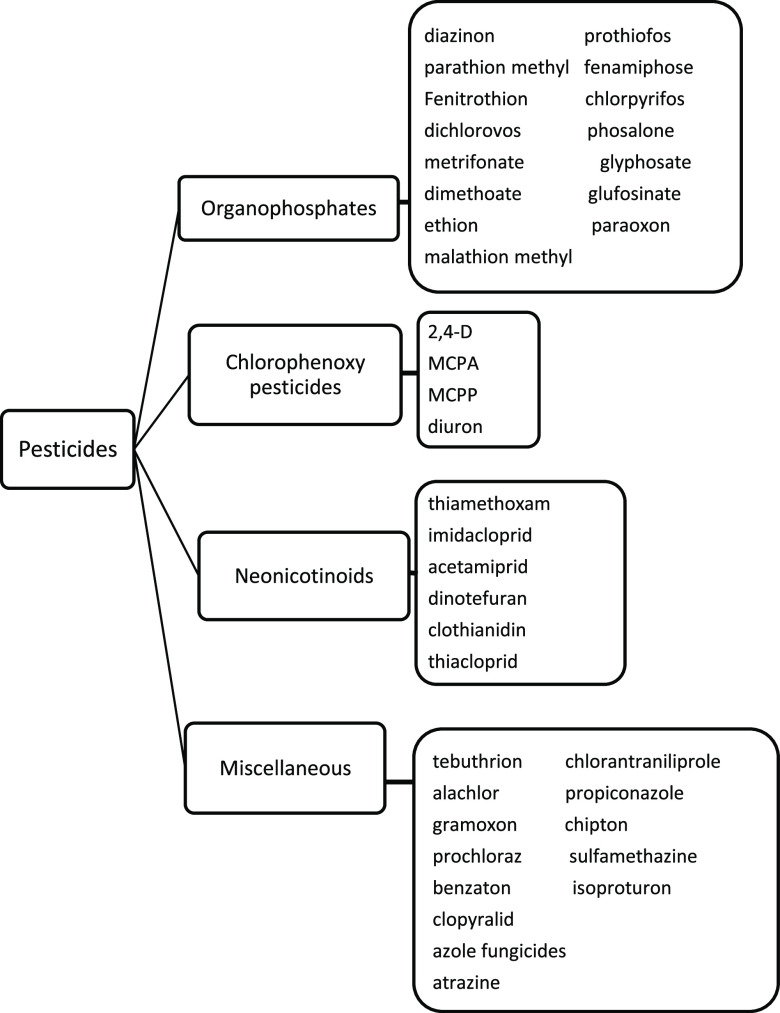
Classification of pesticides used for extraction in this review.

Contrary to the limited number of available studies for the use of MOFs for agrochemical delivery, extraction of pesticides from the environment using MOFs was studied extensively. Effective adsorption bears multiple factors at play, including surface area and pore volume. The fine-tunable properties of MOFs can provide us with good options for enhanced adsorption, from the selection of metal centers, organic linkers, defect engineering to surface modifications. As we will see in the following literature, adsorption can be enhanced by functionalization into nanocomposites that greatly enhance the adsorption capacity by increasing the surface area and adsorption sites. In the following section, the studies are grouped according to the class of agrochemicals extracted (Figure 8). A number of mechanisms for adsorption were proposed, such as electrostatic forces, acid–base interactions, hydrogen bonding, and π–π interactions.

### 3.1 MOFs for Extraction of Organophosphates

Organophosphates (also known as phosphate esters) are a large class of pesticides. This group of insecticides functions by inhibiting acetylcholinesterase enzymes, due to their analogous structure to acetylcholine, an essential neurotransmitter for the communication of neural signals. In addition, for its use as herbicide, this class of effective pesticides presents extreme danger to humans, fauna and flora, and hence due to their high risk and popularity, there are a large number of studies on potential use of MOFs for their removal from the environment. The studies that utilize MOFs for extraction of organophosphates are summarized in Table 2.

**Table 2:**
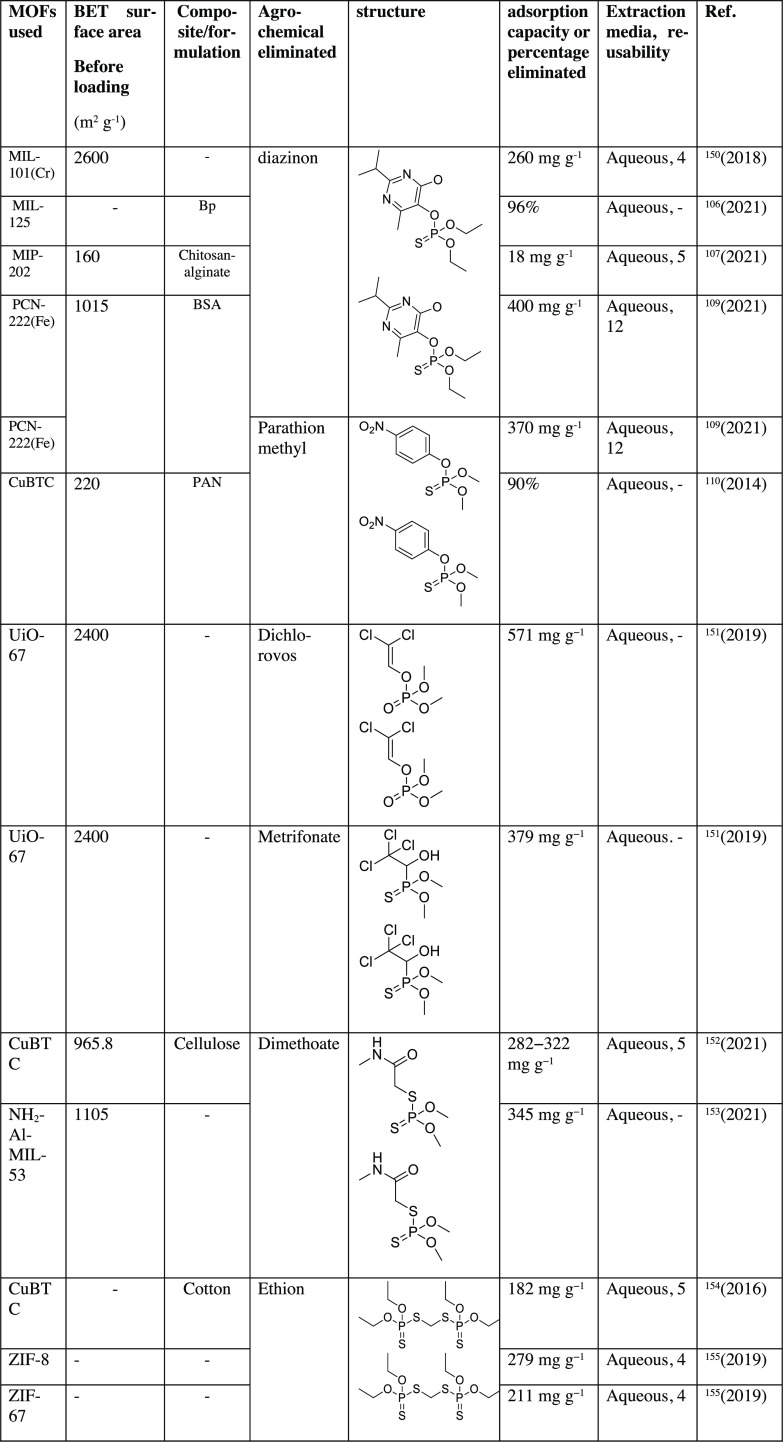

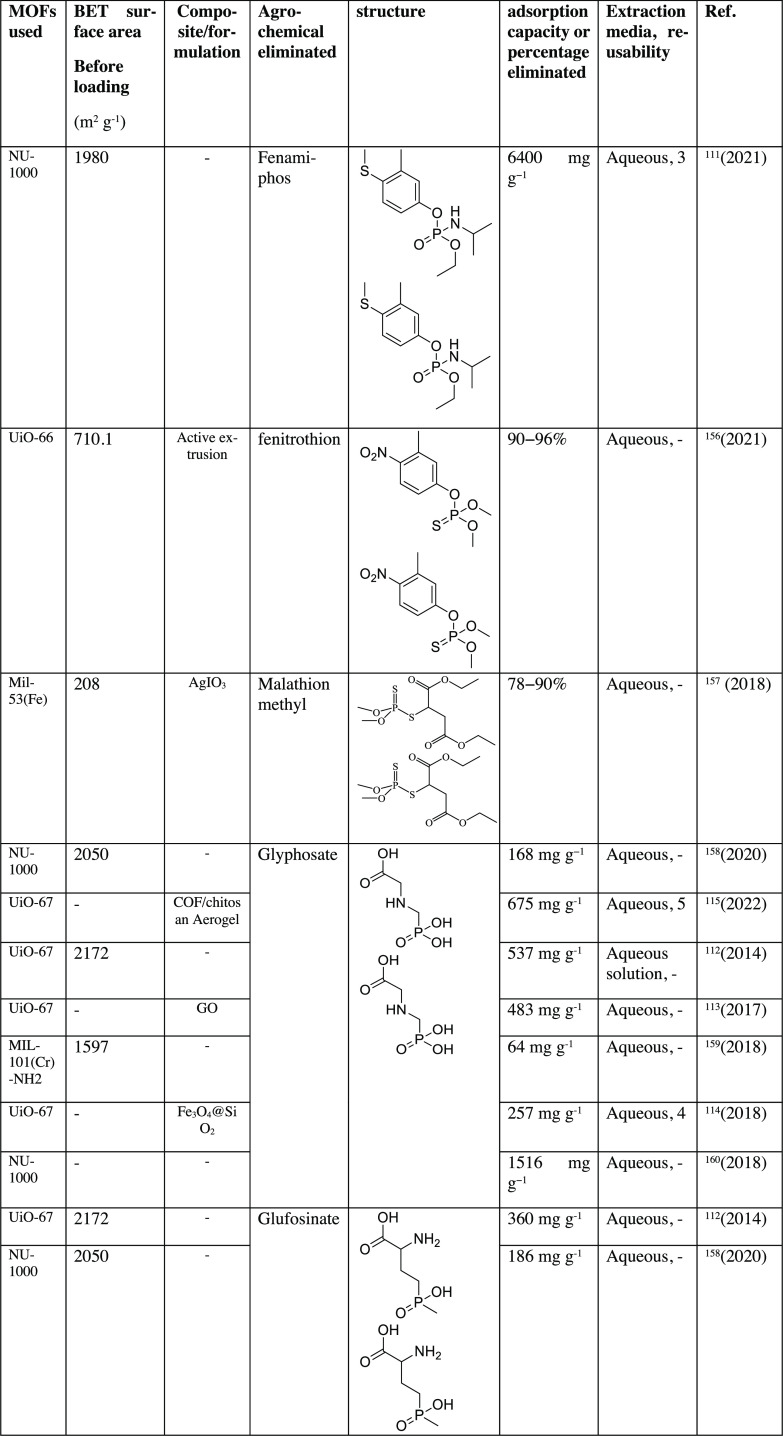

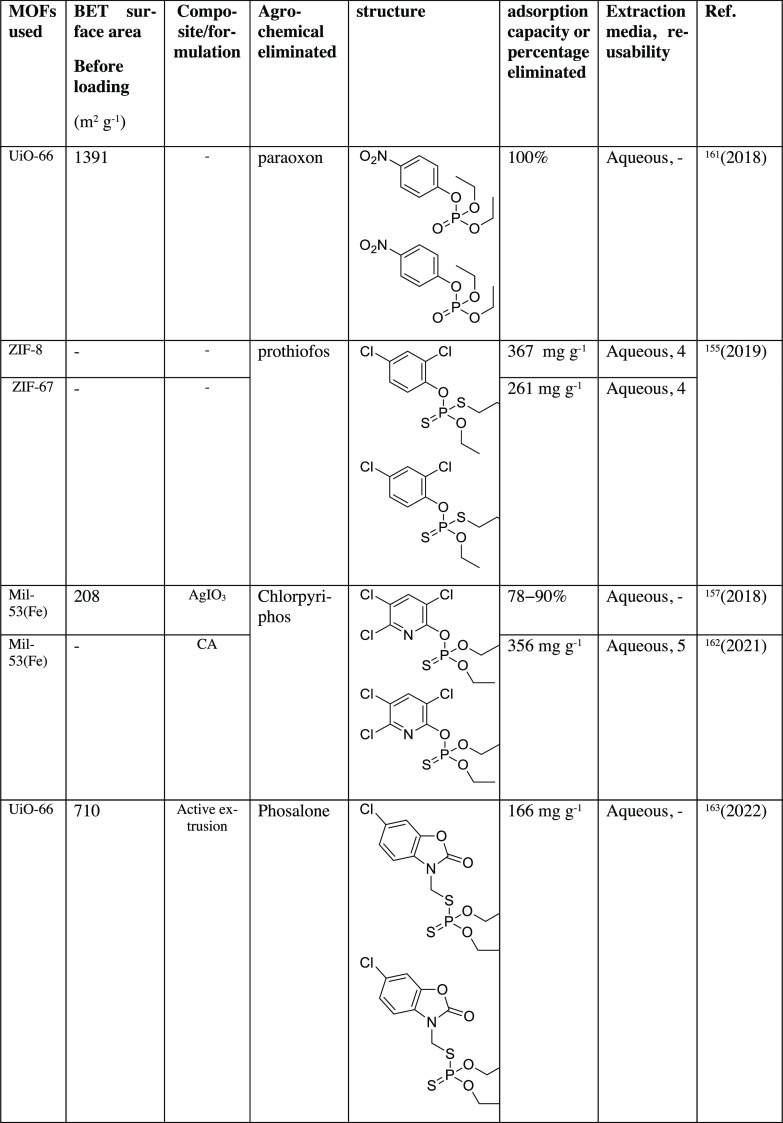
List of Organophosphate Pesticides Extracted Using MOFs^106,107,109,110,111,112,113,114,115,150,151,152,153,154,155,156,157,158,159,160,161,162,163^

In 2018, the organothiophosphate, diazonin, was used as a model insecticide for its extraction from aqueous media using MIL-101(Cr) by continuous fixed bed system,^104^ where the solution to be filtered traveled through a bed of MOF from one end, with the filtrate exiting into a UV-spectrometer from the other end. The efficacy of adsorption seemed to be lower in acidic media, this was postulated to be because of damage to the MOFs. Adsorption efficacy was found to be best at a pH of 6.5. This may be because chromium-based MOFs were proven to catalyze the hydrolysis of heterocyclic phosphate esters.^105^ It was proposed that diazonin could donate the lone pair of electrons on the sulfur atom facilitating complexation with uncoordinated metal ion centers, enhancing its adsorption into the MOF at neutral pH. The reusability of MOFs was studied, proving that acetone was a better solvent at washing of pesticides for regeneration of the MOFs, with only a minor decrease in adsorption efficiency of 0.51% in the second run of adsorption.

Other studies on the adsorption of diazonin in aqueous media^106^ have also determined neutral pH to be optimum for adsorption. In a study by Hlophe et al., MIL-125(Ti) was used as a composite of black phosphorus (BP) for the collection and photocatalysis of diazonin. MIL-125(Ti) showed excellent photoactivity. However, there were some limitations due to charge recombination and activation being in UV region only, and hence, the incorporation of BP composite helped to avert this hindrance in efficacy. As we have seen in the previous studies, acidic conditions cause the degradation of many MOFs rendering them useless. In alkaline conditions, it was determined that both the adsorbent and the adsorbate possess negative charge, and hence decreasing the efficacy of adsorption due to electrostatic repulsion. Optimum conditions were determined to be 4% by weight of BP composition. In neutral pH, 96% removal of diazonin was achieved after 30 min. In addition, effectiveness in photocatalysis increased by 4% when incorporating BP into the MOF as a composite, when compared to the efficacy of the MOF alone. In another study,^107^ the zirconium-based MOF MIP-202 was incorporated with chitosan alginate (CA) to form biobead composites for the adsorption of diazonin from polluted water. Incorporating the MOF into a CA composite greatly enhanced the adsorption capacity when compared to CA, by increasing the sites of adsorption available. The reusability of the MOF composites was tested, and it was found that the capacity declined to 54% after the fifth adsorption cycle. This was a result of the strong forces of attraction due to the presence of excess hydroxide and amine groups on the surface of the composites. Nonetheless, it proved the excellent potential for reusability to lower costs for real life applications.^108^

Composites of iron-based porphyrin MOF, PCN-222, were investigated for the extraction of two organophosphates, diazonin, and methyl parathion in water.^109^ The MOF was coupled with bovine serum albumin (BSA). BSA contains nearly 21 coordination sites in addition to being also a component of human blood serum. The study investigated various effects such as pH, amount of MOF used, volume of solution, and reusability for the extraction of both organophosphates. Like in the previous studies mentioned, the optimum pH for the adsorption of diazonin was found to be 7, with a maximum adsorption capacity of 400 mg g^–1^. The functionalization of PCN-222 with BSA introduced multiple extra binding sites when compared to the MOF alone. This resulted in enhancement of the adsorption capacity, as was observed with MIP-202/CA in the former study mentioned. The preferable enhancement of adsorption is attributed as a mechanism at play, in addition to the excess variety of functional groups acting as binding sites. Defective PCN-222 can form mesochannels as a result of missing linkers, thus exposing unsaturated metal sites and, hence, driving the organophosphates into the pores, facilitated by coordination bonding, van der Waals, electrostatic, hydrogen bonding interactions, and π–π stacking. Additionally, acid–base interaction in the case of diazonin and methyl parathion both showed best extraction at pH 7. This is due to the protonation or deprotonation of different functional groups. The reusability of the MOF composites was tested. The MOF composites were recycled and regenerated 12 times, reaching a reduction of 20% of efficiency for the last round of adsorption.

In 2014, methyl parathion was also extracted from hexane using HKUST-1.^110^ The MOFs were incorporated into polyacrylonitrile (PAN) to form a fiber mat for agrochemical extraction, with the proposed mechanism suggested to be partitioning of methyl parathion into HKUST-1 pores, as a result of the relative solubility. Interestingly, Lange et al. suggested the functionalization of such fiber composites for the slow release of pesticides, before the first account of the use of MOFs for pesticide delivery by Yaghi et al. in 2017.^82^

Fenamiphos, another organophosphate with the structure shown in Table 2, was extracted using NU-1000.^111^ In this study, simultaneous adsorption of fenamiphos and phosphate from water was studied. Optimum adsorption capacity for the pesticide is very high, 6400 mg g^–1^, reaching equilibrium in 120 min. In contrast, the adsorption of phosphate was rapid, reaching saturation in 20 min. The selective recovery and desorption of adsorbates were demonstrated in this study.

**Figure 9:**
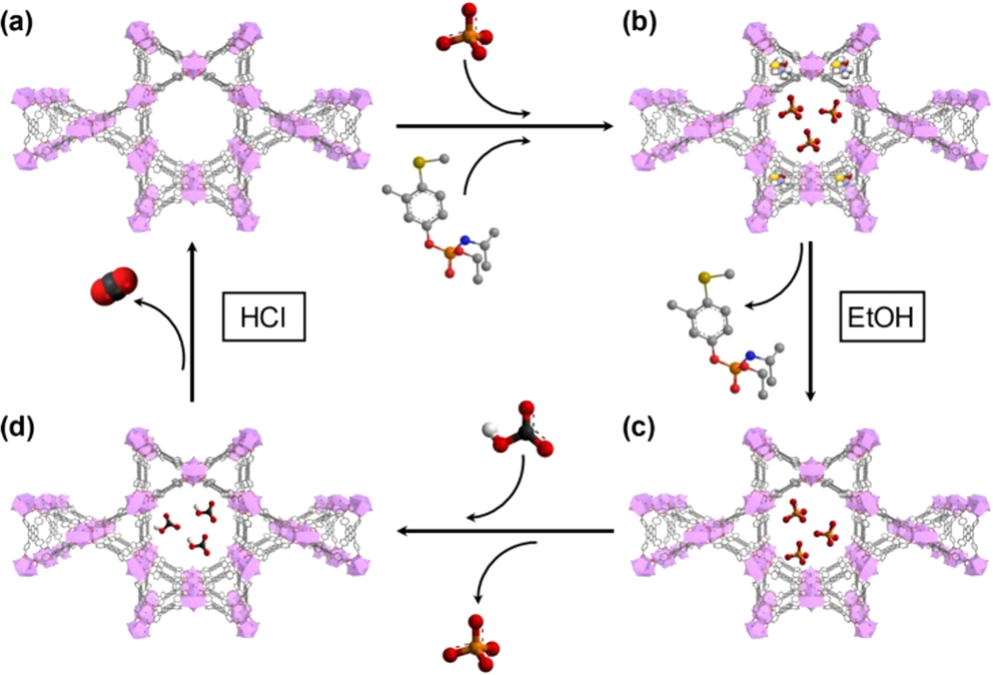
Adsorption–desorption mechanism and selective recovery of fenamiphos. (a) Simultaneous adsorption of phosphate and fenamiphos. (b) Extraction of fenamiphos with ethanol. (c) Ion exchange of phosphate using bicarbonate. (d) Regeneration of the MOF (NU-1000) by treatment with HCl. Reproduced and adapted with permission from ref (111). Copyright 2021 Elsevier.

For the selective recovery, regeneration of NU-1000 was performed in three steps: first, selective desorption of fenamiphos was carried out by suspending loaded MOFs in ethanol, followed by solvent exchange with hydrogen carbonate to remove phosphate (Figure 9). Finally, HCl was used to remove hydrogen carbonate inside the MOFs. This adsorption–desorption method showed 100% reusability of the MOFs with exceptional selectivity for extraction and release. Molecular modeling calculations have confirmed the capability of bulky organophosphates, like fenamiphos, to adsorb into the MOFs, showing favored adsorption for triangular and hexagonal pore shapes. Fast diffusion and unsuccessful desorption attempts with water suggest adsorptive interactions involving van der Waals interaction and π–π stacking with the pore walls.

Glyphosate (GP) and glufosinate (GF), two very popular organophosphate herbicides, were extracted from polluted water by using the Zr-based MOF, UiO-67.^112^ Several factors influencing the adsorption were studied, including concentration of pesticide, pH, amount of MOF added, and ionic strength. Maximum adsorption was found at pH of 4 for GP and 5 for GF. Measurements for the zeta-potential showed that electrostatic forces between the adsorbent and the adsorbates were negligible, and that the force driving the herbicides into the MOF pores is due to chemisorption rather than electrostatic adsorption. Adsorption was found to decrease at pH > 7, due to the negative charge forming on MOF, as well as the electronegative organophosphates. The adsorbent dose was found to be inversely related to uptake capacity. The adsorption capacity at optimum conditions was 537 mg g^–1^ and 360 mg g^–1^ for GP and GF, respectively. When comparing the two herbicides, GP showed a higher affinity for adsorption and a higher *q*_max_, which was proposed as a result of increased ability to bond with Zr–OH groups, when compared to that of GF which has a methyl group present on the phosphorus.

In another study, UiO-67 was incorporated into graphene oxide (GO) composites for the extraction of GP from water.^113^ Like in the previous study mentioned, the optimum pH is 4, with an adsorption capacity of 482 mg g^–1^. When comparing the adsorption kinetics of UiO-67 compared to UiO-67/GO composite, both displayed two-step adsorption. First, a rapid adsorption stage, due the plenty of available adsorption site, and a slow adsorption stage, where the rate of adsorption starts to decrease gradually, until adsorption stops, due to the impediment of bound GP on the adsorption of more GP. Adsorption can be best described as pseudo-second-order, as the main mechanism of adsorption is chemisorption rather than electrostatic. Kinetic parameters show a higher adsorption rate for UiO-67 with a *k*_2_ value of 0.0342 min^–1^, while GO-modified UiO-67 had a *k*_2_ of 0.0030 min^–1^. However, UiO-67/GO had better adsorption rate and loading capacity compared to that of GO, suggesting that UiO-67 is responsible for most of the adsorption.

Another study by Yang et al.^114^ also reported UiO-67 for the extraction of GP, by modifying the MOFs into magnetic nanocomposites of Fe_3_O_4_ and SiO_2_. Glyphosate was extracted from water by magnetic solid phase extraction (MSPE) where the adsorbent particles would be separated by an external magnetic field. Like the previous studies mentioned above, optimum pH for adsorption was found to be 4, due to higher affinity of glyphosate to the Zr–OH sites in acidic pH. Kinetic studies also proved that the adsorption was closely resembling pseudo-second-order, with adsorption capacity of 256.54 mg g^–1^. The composites showed good performance for recycling over four cycles before showing a significant decrease in adsorption capacity. Desorption was done in a solution of 28% ammonia at a pH of 10 to release GP. Magnetization of such composites with Fe_3_O_4_ proved useful for recyclability, providing an efficient and convenient method of separation.

**Figure 10:**
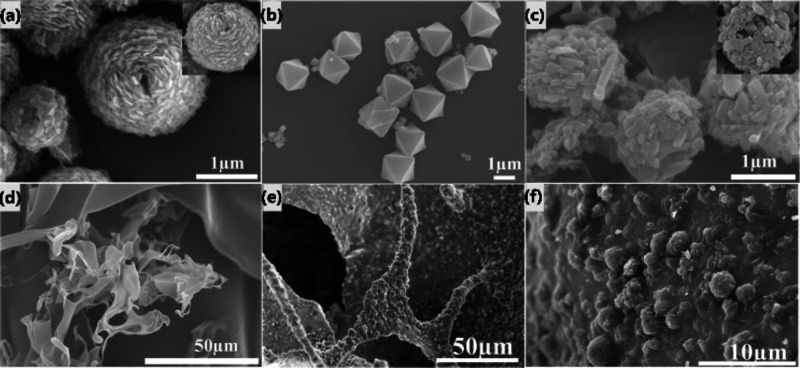
SEM images of the MOF-on-COF chitosan composites of (A) pristine MCA, (B) UiO-67, (C) MCA/UiO-67, (D) pure CS aerogel, and (E,F) CS-MCA/UiO-67. Reproduced and adapted with permission from ref (115). Copyright 2022 Elsevier.

In 2022, Luo et al. investigated the extraction of GP from aqueous media using the hybrid MOF-on-COF composites, CS-MCA/UiO-67,^115^ where the Zr-based MOF, UiO-67, was loaded onto a covalent–organic framework (COF) in situ. This functionalization gave rise to a much higher surface area for the adsorption of pesticides. Further modification with chitosan aerogel also allowed for quick diffusion of the herbicide into the composite. The composites exhibited coral-like structures, as shown in Figure 10.

The composites showed a high adsorption capacity of 675 mg g^–1^ for GP. The functionalization of UiO-67 with a COF and the chitosan aerogel created an enhanced number of sites for the higher adsorption. The composites could be reused five times without showing a degradation in adsorption capacity.

In 2018, Lui et al. used ZIF-8, functionalized with magnetic multiwalled carbon nanotubes, as a magnetic sorbent for the extraction of organophosphates from water,^116^ showing 95% elimination from water and soil samples.

In this section, we have given two examples of organophosphates: phenolic organophosphates, like fenamiphos and diazonin, and aliphatic organophosphates, like glyphosate and glufosinate. In Table 2, we have summarized the rest of available literature harnessing MOFs for the extraction of organophosphates.

**Figure 11:**
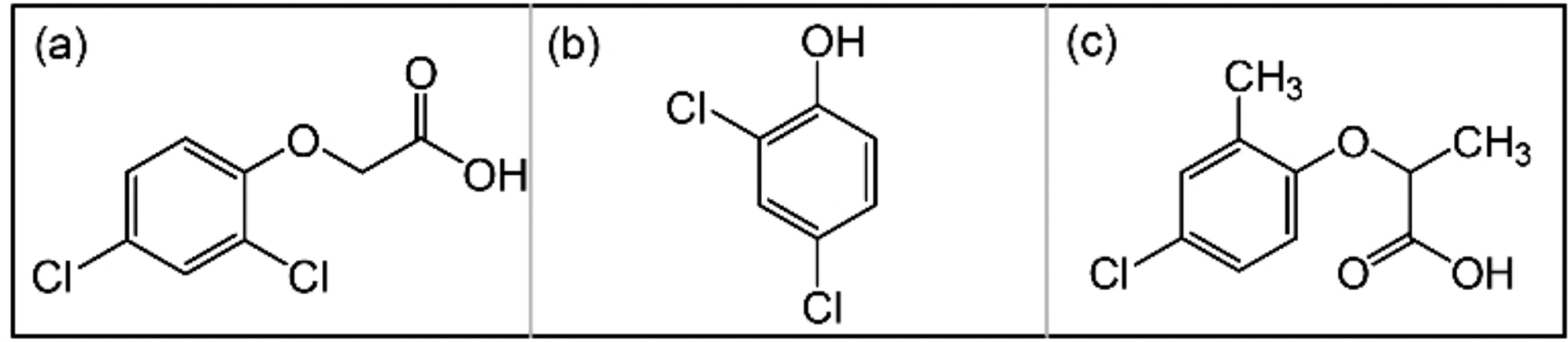
Chemical structures of common chlorophenoxy herbicides of (a) 2,4-D, (b) 2,4-DP, and (c) mecoprop (MCPP).

### 3.2 MOFs for Extraction of Chlorophenoxy Herbicides

**Figure 12:**
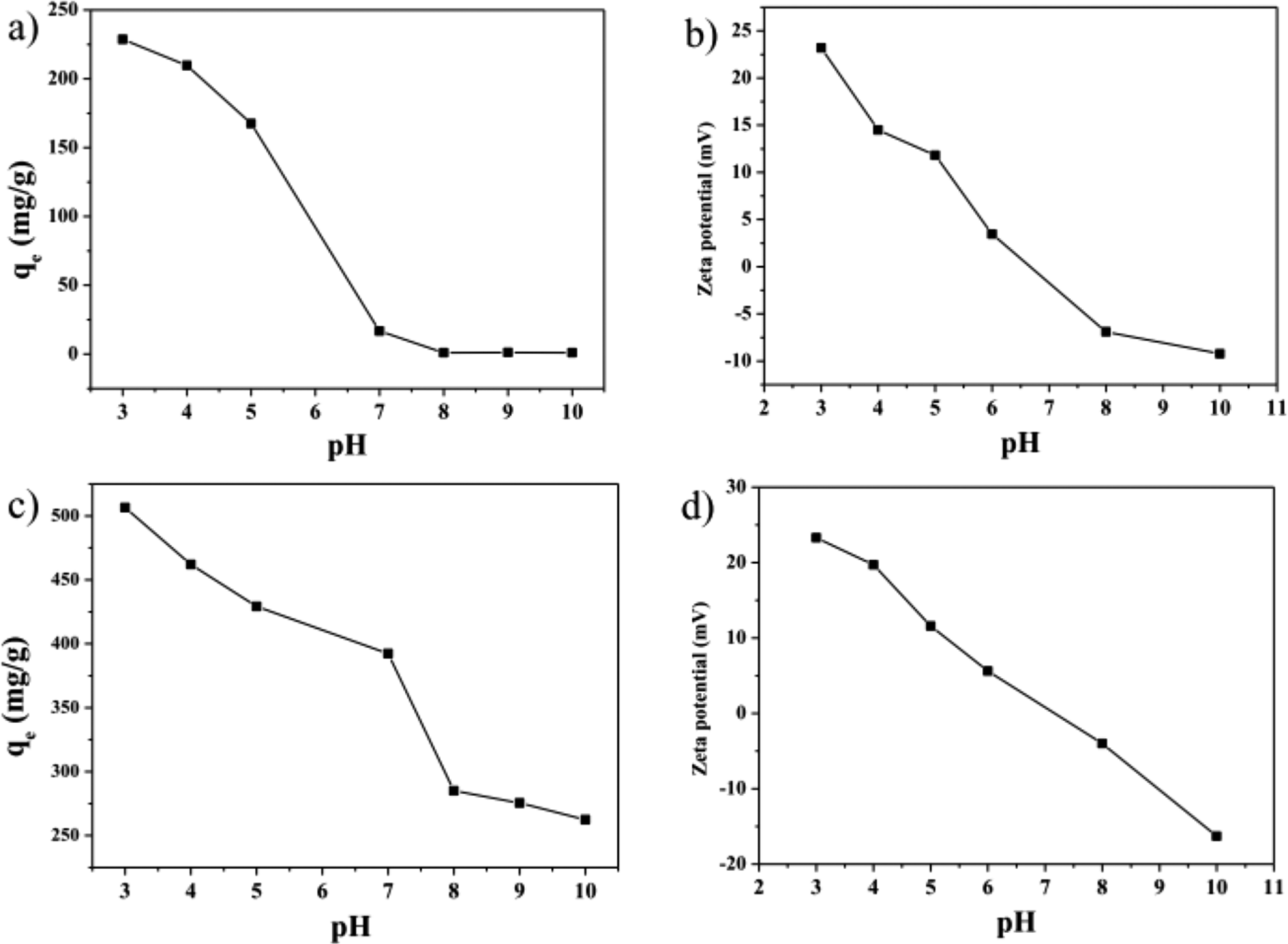
pH-Dependent change in loading capacity at equilibrium, *q*_e_, and the zeta-potential for (a,b) Mil-101(Cr) and (c,d) Mil-101(Cr)-Cl. Reproduced and adapted with permission from ref (117). Copyright 2017 under the Creative Commons Attribution License.

Chlorophenoxy herbicides are a class of pesticides used for the selective killing of broadleaf weeds (Figure 11). In 2017, Chen et al. utilized cationic MOFs for removal of 2,4-dichlorophenoxyacetic acid (2.4-D) from water.^117^ The chromium-based MOF, Mil-101(Cr)-Cl, with a BET surface area of 3932 m^2^ g^–1^, is functionalized with Cl^–^ as a mobile ion. In a typical synthesis of MIL-101(Cr), HF is used as a mineralizing agent for better crystallinity.^118^ To modify the neutral MOF into a cationic one, MIL-101(Cr)-Cl is prepared by postsynthetically exchanging F^–1^ with Cl^–1^. Adsorption kinetics showed a high initial adsorption rate, due to the available Cl^–1^ ions to be replaced, as well as the presence of the positive charge on the surface of the MOF. With time, the adsorption rate decreases gradually, due to the decrease in the positive charge of MIL-101(Cr)-Cl, as well as the obstruction caused by the adsorbed 2,4-D in the pores. When comparing MIL-101(Cr) to the cationic MIL-101(Cr)-Cl, the latter has an adsorption capacity for 2,4-D greater than that for MIL-101(Cr) in the same conditions. This is due to the effect of anion stripping on the adsorption mechanisms.^119^ The adsorption capacity of 2,4-D decreased for MIL-101(Cr) at high pH due to the repulsive electrostatic interactions. However, this pH dependent decrease did not occur for MIL-101(Cr)-Cl. In fact, the adsorption capacity was much higher at pH 7 for MIL-101(Cr)-Cl, as shown in Figure 12, due to the ion-exchange adsorption process.

Another cationic MOF, UiO-66-NMe_3_^+^, was synthesized and used for investigating the adsorption of 2,4-D from aqueous media.^120^ The quaternary amine MOF is synthesized using UiO-66-NH_2_ as a starting material followed by N-methylation by reacting with methyl triflate in dichloromethane solution. The BET surface area was measured to be 509 m^2^ g^–1^, which is lower than that of UiO-66 and UiO-66-NH_2_, as a consequence of the quaternary methylated amine group occupying the space. Adsorption capacity was observed to be highest at a pH of 2. The driving forces of adsorption were attributed to electrostatic forces, π–π stacking, hydrogen bonding, as well as ion exchange. The effect of the presence of other anions on the adsorption capacity was also investigated, as they might compete with 2,4-D anions to occupy the positively charged adsorption sites. In general, it was noted that the presence of smaller monovalent anions, like chlorine, tend to have the largest decrease in the adsorption capacity, due to their ability to quickly occupy the binding sites, in contrast to bulky divalent anions like sulfate, having hindered accessibility to adsorption sites, as a result of their tetrahedral structure.

Adsorption kinetics show that equilibrium is reached at 120 min. Adsorption behavior seemed to better fit the pseudo-second order model indicating that the adsorption is primarily relied on chemisorption. This might be explained due to the presence of 2,4-D as an anion, interacting with the cationic adsorption sites. In addition, Lewis acid–base reactions might occur between hydroxyl groups from the oxo-zirconium sites and the electronegative chlorine atom in the 2,4-D structure.

UiO-66 and UiO-66-NH_2_ were used for the adsorption of 2,4-D.^121^ Adsorption experiments were carried out in aqueous solution, and various effects like pH and adsorbent dosage on the adsorption capacity were studied. For dosage, as the amount of MOF increased, the amount of 2,4-D captured increases, due to the increased number of active sites available for adsorption, reaching the highest adsorption capacity of 350 mg g^–1^, and a removal rate of 95% was achieved. UiO-66-NH_2_ had better adsorption than UiO-66, due to the presence of amine groups. The effect of pH on adsorption capacity is dependent on the physical and chemical characteristics of both the adsorbent and the adsorbate. 2,4-D has a p*K*_a_ of 2.8 and exists as anions in pH > 3. For UiO-66-NH_2_, the zeta-potential at pH < 6.2 is positive, thus exhibiting a positive charge on the MOF surface. Additionally, at pH 10, the adsorption capacity decreased dramatically, due to maximal electrostatic repulsion between 2,4-D and the MOFs. Hence, the optimum pH for adsorption ranges from 3 to 6, due to the presence of electrostatic attraction, as well as hydrogen bonding and π–π stacking for UiO-66-NH_2_ and UiO-66, respectively. Experiments on the reusability were performed by suspending the 2,4-D loaded MOFs in a 0.01 mol L^–1^ solution of NaOH, to exchange 2,4-D with OH^–^. The MOFs proved to be reusable for five cycles before reaching a removal efficacy of 95% after the fifth use.

**Figure 13:**
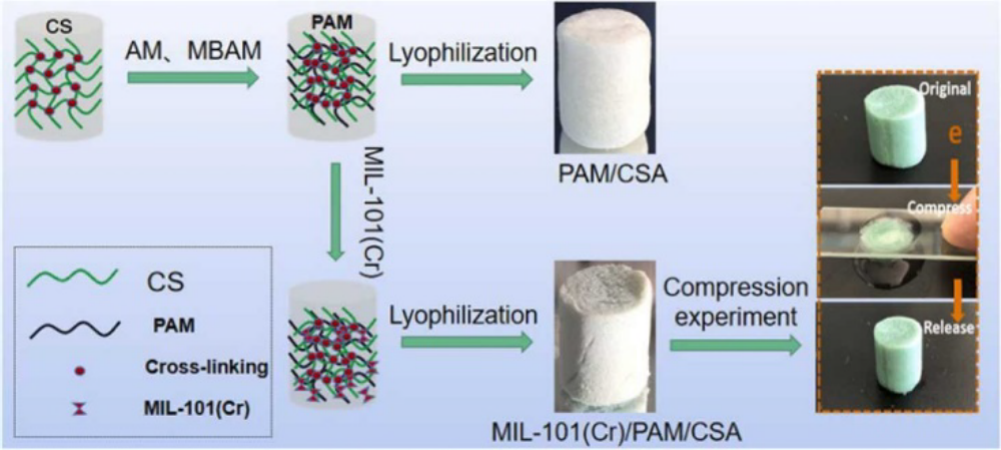
MIL-101(Cr)/PAM/CSA composition, as well as its elastic ability. Reproduced (including photo) and adapted with permission from ref (122). Copyright 2022 Elsevier.

Another study for the adsorption of 2,4-D from water utilizes a super elastic MIL-101(Cr) MOF composite at a 30% weight MOF composition.^122^ The composite is formed from polyacrylamide (PAM) and chitosan aerogel (CSA) that can be regenerated by compression, as shown in Figure 13, followed by washings with methanol, distilled water and freeze-drying. Similar to the other studies discussed above maximum adsorption capacity for 2,4-D was found at pH of 4. The adsorption capacity of MIL-101(Cr) was measured to be 161 mg g^–1^ proving to be a good adsorber of 2,4-D. The incorporation of MOF to the polymer structure, PAM/CSA with a BET of ∼58 m^2^ g^–1^, improved its adsorption capacity from 118 mg g^–1^ to 153 mg g^–1^ in addition to increasing its BET surface area to 311 m^2^ g^–1^. This is a result of the increase in adsorption sites as well as the mesoporous and microporous structure of the MOF.

The mechanisms of adsorption were discussed, and the trend follows the previous studies mentioned on the adsorption on 2,4-D in this review so far. Factors like zeta-potential and the p*K*_a_ of the adsorbent and the adsorbate explains the mechanisms of adsorption as well as the optimum conditions for maximum adsorption capacity. Possible mechanisms for adsorption are attributed to electrostatic attraction, π–π stacking and hydrogen bonding, with the PAM/CSA rich in amine groups. Regeneration of the composites showed an efficacy of 93% despite repetitive reuse. The mechanical properties of such elastic composites provide a greener method for reusability, as most of the contents adsorbed would be expelled upon compression, thus requiring less resources for washing for the purpose of reusability.

In 2020, Li et al. reported UiO-66-NH_2_ sponges for the extraction of 2,4-D from water.^123^ The synthesis of the composite, UiO-66-NH_2_/sponge, was performed by the immersion of a nitrile butadiene rubber of dimensions (1 × 1 × 0.5 cm^3^) in the precursor solutions of UiO-66-NH_2_, which was then transferred to a Teflon-lined stainless steel reaction container and left for 24 h to react.

**Figure 14:**
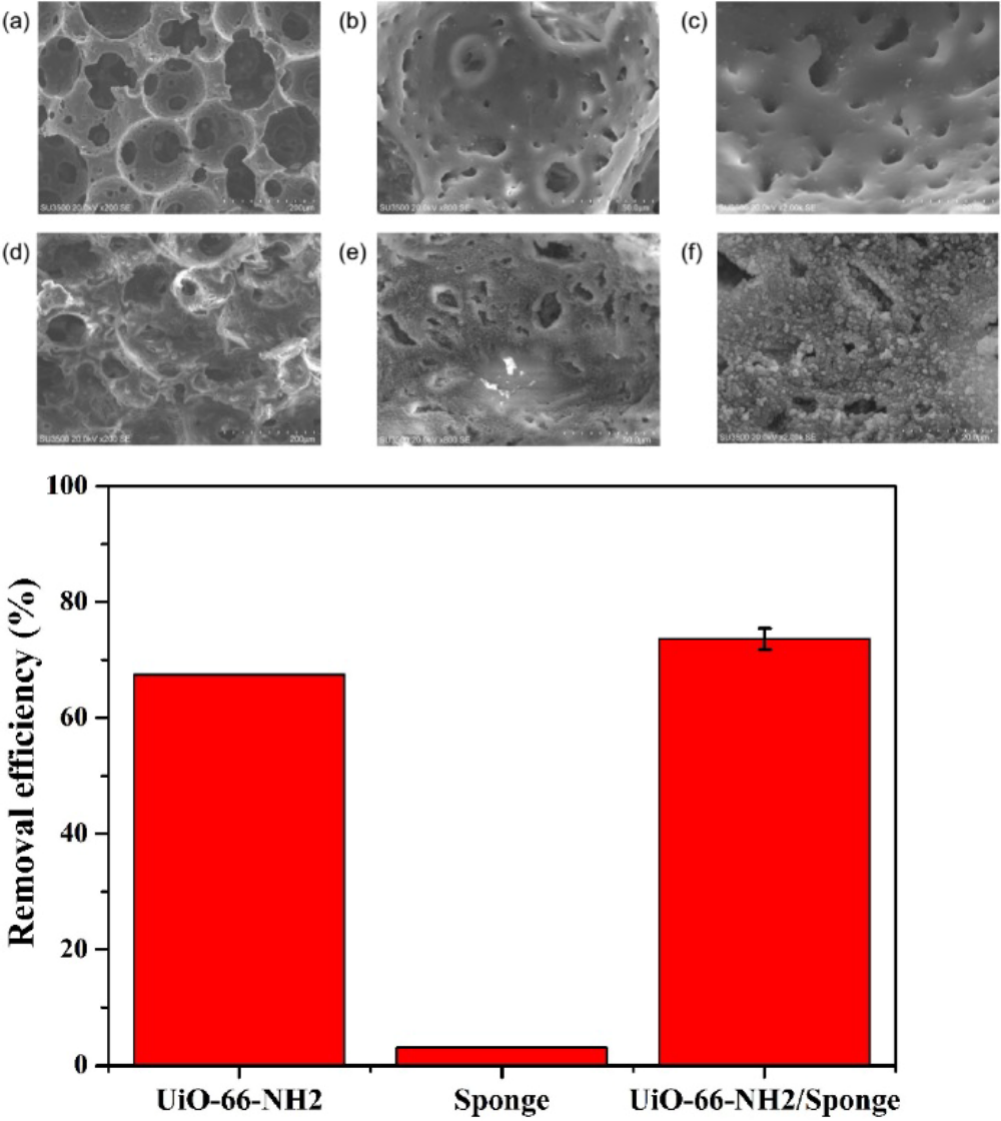
Top: SEM images of (a–c) nitrile butadiene rubber and (d–f) UiO-66-NH_2_/sponge. Bottom: Removal efficacy of UiO-66-NH_2_, nitrile butadiene sponge, and UiO-66-NH_2_/sponge. Reproduced and adapted with permission from ref (123). Copyright 2020 Elsevier.

Adsorption studies were performed on the sponges. The conditions were optimized for maximal adsorption capacity, and best performance was observed at pH 3. Mechanisms of adsorption were proposed to be, π–π stacking, electrostatic attraction, and hydrogen bonding, between 2,4-D and −COOH and −NH_2_. Adsorption kinetics fit well with pseudo-second order, and the calculated model were in good agreement with the experimental data. Adsorption isotherms show an initially increased adsorption capacity as the concentration of 2,4-D is increased, reaching equilibrium. The maximum adsorption capacity was measured to be 72 mg g^–1^ with a good fit to the Langmuir model, indicating a monolayer adsorption at a limited number of similar binding sites. When comparing the adsorption efficacy, it was observed that the incorporation of UiO-66-NH_2_ greatly enhances the efficacy of the nitrile butadiene sponge, as shown in Figure 14. The regeneration ability proved its good recyclability over three test cycles, before showing a decrease in the adsorption efficacy. Multiple washing with ethanol showed the lowest decrease in the adsorption capacity, proving the strong forces binding 2,4-D to the composite.

In an interesting study done by Isaeva et al. in 2019, the MIL-53^124^ was investigated for the extraction of 2,4-D from water. This MOF can undergo reversible transformations for its framework between open, closed, narrow, and large pore sizes. This structural change is called a “breathing effect”, and it allows for stimuli triggered adsorption, and expulsion of adsorbates. In amine functionalized MIL-53(Al), the proposed mechanism for this effect is attributed to the hydrogen bonding between the – NH_2_ group of the linker, and uncoordinated oxo-metal sites, this gives rise to fine-tuning property, helpful for adsorption. In this study, mixed linkers were used for the synthesis of three MIL-53(Al)-based MOFs, by in situ modification of 1,4-dicarboxylic acid and 2-aminobenzene-1,4-dicarboxylic linker ratios in three proportions. Adsorption capacities of NH_2_-MIL-53(Al) were the lowest, at 250–240 mg g^–1^, when compared to those of other linker ratios, due to the maximal NH_2_ presence causing a decrease mesoporous when compared to MIL-53(Al) and mixed linker variants. Lower flexibility of the NH_2_-MIL-53(Al) was observed compared to MIL-53(Al), and the adsorption capacity was measured for 225 h without any noticeable effect on performance and crystal structure.

Luo et al. also investigated the adsorption of 2,4-D using the composite CS-MCA/UiO-67.^115^ The composites showed high adsorption capacity of 2,4-D, with a capacity of 615.12 mg g^–1^ reaching equilibrium at 60 min.

In another study, Tan et al. used the iron-based MOF, MIL-100(Fe) for the remediation of 2,4-D.^125^ Adsorption was shown to be rapid, reaching 50% of adsorbed pesticide in 5 min, reaching a maximum adsorption capacity of 858 mg g^–1^. Mechanisms of adsorption was primarily attributed to electrostatic attraction. In addition, kinetic studies indicated a pseudo-second order for the adsorption process. The MOF was recycled 5 times without showing a significant change in adsorption capacity.

In another study, 2-methyl-4-chlorophenoxyacetic acid (MCPA) was studied for its extraction from aqueous solution by the amine functionalized Zr-MOF, UiO-66-NH_2_.^126^ Kinetic studies show very rapid adsorption within the first 3 min, accounting for most of the adsorption capacity. Adsorption kinetics could be fit with a pseudo-second order model, indicating chemisorption as the rate limiting step. The highest adsorption capacity was found to be 300 mg g^–1^. The proposed mechanism of adsorption is due to hydrogen bonding, π–π stacking, and electrostatic attraction with MCPA and UiO-66-NH_2_. When compared to UiO-66, the amine-modified MOF had a much higher adsorption, as a result of the involvement of the amine group in enhancing the attraction forces between MCPA and UiO-66-NH_2._ The MOF also demonstrated excellent reusability, and was recycled at least 6 times without showing a noticeable loss in adsorption capacity. The photodegradation of 2,4-D and MCPA from polluted water using the silver based Ag-MOF.^127^ In this study, Hayati et al. demonstrated that an Ag-MOF showed excellent photocatalytic activity against 2,4-D and MCPA, with up to 96 and 98% efficiency at optimum conditions, respectively. The MOFs also demonstrated excellent reusability in terms of efficiency, with a decrease to 86% for the fifth cycle.

Another chlorophenoxy herbicide, methylchlorophenoxypropionic acid (MCPP), was extracted from aqueous media by Seo et al.^128^ using UiO-66. The adsorption kinetics was compared to activated carbon, showing that UiO-66 had a much higher adsorption rate. Mechanisms of adsorption were proposed as electrostatic forces and π–π stacking. The MOFs also demonstrated their ability to be reused after simple washing with water and ethanol.

In another study, MOF Derived Carbon (MDC) was used for the extraction of Duiron and 2,4-D.^129^ It was synthesized from the pyrolysis of ionic liquid (IL)-loaded ZIF-8. IL was prepared by a ship-in-bottle technique inside ZIF-8 pores. Although the incorporation of IL decreased the surface area, it enhanced the capacity and rate of adsorption when compared to MDC derived from ZIF-8 without IL. This can be explained by the abundance of nitrogen and oxygen groups from the IL aiding in adsorption. The adsorption capacity was 284 and 448 mg g^–1^, for DCMU (3-(3,4-dichlorophenyl)-1,1-dimethylurea) and 2,4-D, respectively. The IL-functionalized MDC showed its ability to regenerate for reusability with simple washing with solvent.

### 3.3 MOFs for Extraction of Neonicotinoids

Neonicotinoids are a class of insecticides with a similar structure to nicotine. They account for about 30% of the total worldwide sales of insecticides.^130^ Despite their popularity, there have been extensive regulations and banning their applications.^131^ Some of the available neonicotinoids in the market are thiamethoxam, imidacloprid, acetamiprid and dinotefuran.^132^

In 2016, Cao et al. investigated the extraction of 7 neonicotinoid pesticides,^133^ dinotefuran, nitenpyram, clothianidin, thiamethoxam, imidacloprid, acetamiprid, and thiacloprid, from water samples using a magnetic MOF composite, MOF-199/Fe_3_O_4_. The composites were synthesized in situ, where Fe_3_O_4_ was added to the reaction mixture containing copper(II) acetate and benzene-1,3,5-tricarboxylic acid (H_3_BTC). The resultant copper MOF composite was then used for extraction of the pesticides. Adsorption mechanisms were explained to be due to acid–base interactions, hydrophobic interactions, and π–π stacking. In general, π–π stacking contributed the most to the adsorption, as a result of the benzene ring interacting with the delocalized π-bonds of the insecticide molecules.

Thiamethoxam was studied for adsorption and catalytic degradation using MIL(Fe)/Fe-doped nanospongy porous carbon composites.^134^ The porous carbon (Fe-SPC) is derived by the treatment of silkworm excrements with 5 mL of ZnCl_2_ and FeCl_2_ salts in HCl, followed by heating at 800 °C, to create highly porous carbon templates. The composite was then synthesized by in situ incorporation during the synthesis step of Mil-101(Fe).

Thiamethoxam is rapidly catalyzed into a stable intermediate and adsorbed into the MOF via strong chemisorption. The composites also exhibited ultrahigh removal efficiency, with a total organic carbon (TOC) of 95% in 180 min at room temperature.

In 2021, Negro et al. used a thioether based MOFs derived from l-methionine and *S*-methyl-l-cysteine to capture the neonicotinoids clothianidin, acetamiprid, thiacloprid, imidacloprid, and thiamethoxam from aqueous solution.^135^ One MOF, synthesized by combining both amino acids in equal amounts, had an exceptionally high removal efficacy, removing 100% of thiacloprid and acetamiprid in a single step in most conditions. As well as removing about 71–86% for clothianidin, imidacloprid, and thiamethoxam, the MOFs showed excellent reusability over 10 cycles without any change in adsorption capacity.

Another study for the extraction of imidacloprid and thiamethoxam used magnetic porous carbon, derived from ZIF-67 on cornstalk.^136^ The porous carbon was synthesized by first pregrowing ZIF-67 on corn stalk, pyrolyzing, and finally by treating with acid. The pyrolysis process converted the organic linker of the ZIF-67 into graphitic carbon, which captured the inorganic cobalt moiety of the ZIF. The porous carbon showed strong stability against acid and exhibited strong magnetism due to the presence of the Co nanoparticles that was reduced from Co^2+^ during the carbonization process. The porous carbon showed a BET surface area of 280 m^2^ g^–1^. The adsorption capacities of imidacloprid and thiamethoxam reached 189 and 133 m^2^ g^–1^, respectively. In addition, the porous carbon showed excellent recyclability, with an adsorption capacity of 95% after 6 cycles. Mechanisms of adsorption were proposed to be hydrogen bonding and π–π stacking. In addition, the porous carbon was tested to be safe on wheat growth, with dose-dependent mortality selectively toward *Daphnia carinata*.

A copper-based magnetic MOF (M-MOF) nanocomposite was prepared with a coating of magnetic Fe_4_O_3_, graphene oxide (GO), and β-cyclodextrin (β-CD) and used for adsorptive removal of^137^ thiamethoxam, imidacloprid, acetamiprid, nitenpyram, dinotefuran, clothianidin, and thiacloprid from aqueous media.^137^ The nanocomposites showed more favored adsorption capacity toward thiacloprid. Adsorption kinetics have shown pseudo-second-order adsorption for the seven neonicotinoids, suggesting the contribution of chemisorption to most of the adsorption. The primary adsorption mechanisms were proposed as electrostatic interactions, π–π stacking, and hydrophobic interactions, as a result of the delocalized π-electrons of the benzene rings and the heterocycles, hydrophobic, and nitrogen-containing groups of the pesticide.

### 3.4 MOFs for Extraction of Other Miscellaneous Pesticides

Pesticides are composed of a very wide range of chemicals, with varying chemical structures. So, in this section, some of the studies regarding the extraction of other pesticides are discussed.

Yang et al. postsynthetically modified the chromium-based MOF, Cr-MIL-101, by furan/thiophene functionalization.^138^ This functionalization increased the effect of π–π stacking, hence enhancing the adsorption rate and adsorption capacity of the pesticides. The furan-functionalized Cr-MIL-101 exhibited good adsorption capacity of four aromatic herbicides (tebuthrion, alachlor, diuron, and gramoxone) with adsorption capacities of 80, 123, 149, and 50 mg g^–1^, respectively. When Cr-MIL-101 MOFs were functionalized with 2-methyl furan, 2-ethyl furan, thiophene, and 2-bromofuran, it showed enhanced adsorption ranging from 2.2- to 3.8-fold, when compared to that of nonfunctionalized Cr-MIL-101. The 2-bromofuran-modified MOF exhibited the maximum adsorption capacity. In addition, the MOFs showed excellent herbicide removal efficacy of up to 97%.

Another fungicide, propiconazole, was extracted from aqueous using MIL-101(Cr).^139^ The maximum adsorption capacity was 9.78 mg g^–1^, and the adsorption efficacy reached 90% at optimum conditions. In addition, the kinetic studies fit the pseudo-second-order model for adsorption. The reusability of the MOFs was tested over five adsorption cycles, showing a decrease in adsorption efficacy of 21% after the last cycle. It was also shown that washing with ethanol is best for regeneration and reusability of the MOFs.

**Figure 15:**
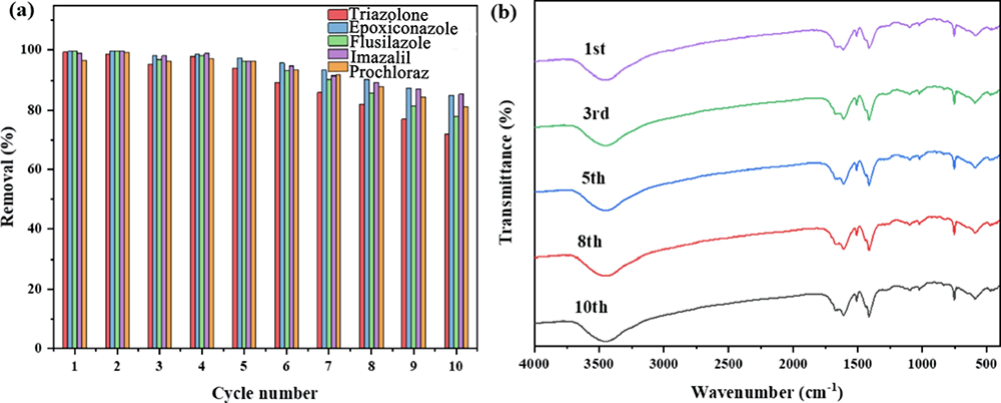
(a) Removal efficiency across 10 cycles. (b) Fourier transform infrared spectra of the composites for 10 adsorption cycles. Reproduced and adapted with permission from ref (140). Copyright 2022 Elsevier.

Prochloraz, another fungicide, was extracted from aqueous solution by Zhou et al. using a composite of magnetic nanoparticles of Fe_3_O_4_, ZIF-90, and MIL-68(AL).^140^ The adsorption capacity was 352 mg g^–1^ at optimum condition. In addition, the adsorption kinetics also fit the pseudo-second-order model. Further investigations on paddy field water samples contaminated with azole fungicides, triadimefon, imazalil, epoxiconazole, flusilazole, and prochloraz were done. As shown in Figure 15, the removal efficiency remained above 90% for all samples, and the composites demonstrated excellent reusability.

Incorporating ZIF-90 into the magnetic MOF composite greatly enhanced the adsorption capacity, increasing the capacity of adsorption from 25 to 352 mg g^–1^, with an increased the surface area from 291 to 392 m^2^ g^–1^. In addition to making the magnetic adsorbent more stable.

Another study reported using magnetic Fe_3_O_4_ MOF composites on azole fungicides have used MIL-53(Al) functionalized with zinc double layered hydroxides (ZnAl-LDH)^141^ and MIL-100(Fe),^142^ with adsorption capacities of 72 and 102 mg g^–1^, respectively.

The herbicide atrazine was examined for adsorption by the zirconium-based MOF, NU-1000.^143^ Adsorption kinetic showed that the uptake of atrazine is rapid, reaching saturation in less than 5 min, with a maximum adsorption capacity of 36 mg g^–1^, However, 98% of the adsorption capacity was reached in 1 min, demonstrating rapid diffusion of atrazine into the mesopores of NU-1000. It was shown that the pyrene-based linker was mainly responsible for this rapid rate of diffusion, as NU-1008, a MOF akin to NU-1000 in terms of surface area but lacking the π-system of NU-1000, extracted only about <20% of the atrazine dissolved.

Atrazine was also extracted from aqueous solution using ZIF-8, UiO-66, and UiO-67.^144^ Both UiO-67 and ZIF-8 showed a removal efficacy of 98%, reaching equilibrium in 2 and 40 min, respectively. UiO-66 on the other hand, exhibited much lower adsorption capacity and very low removal efficacy.

The catalytic degradation of sulfamethazine as a method of remediation was studied by Du et al.^145^ using porous carbon that is derived from MOF pyrolysis. Removal efficacy was tested to be 97% and the porous carbon was proved to be recyclable up to five times, while maintaining its removal efficacy.

In 2015, De Smedt et al. used MOF-235 for the adsorption of bentazon, clopyralid and isoproturon (IPU) from aqueous solution.^146^ The adsorption capacities were 7.15 mg g^–1^, 9.75 mg g^–1^, and 10.00 mg g^–1^, respectively.

In a study done in 2021, Al-TCPP 3-D MOF nanosheets were used for the adsorption of chlorantraniliprole.^147^ The nanosheets showed an adsorption capacity better than that of the Al-TCPP bulk crystals, with a capacity of 372 mg g^–1^ compared to 222 mg g^–1^. This was due to the presence of higher number of active sites available for adsorption, as well as heightened surface area.

Another pesticide, chipton, was extracted from water by UiO-66-NH_2_ carbon nanotube composites.^148^ The functionalization enhanced the recyclability of the MOF, and reduced the risk of secondary contamination of leaching Zr^4+^. Equilibrium adsorption for chipton is 227 mg g^–1^. The composites demonstrated good reusability over 5 cycles.

Fipronil sulfone, an insecticide that belongs to the phenyl pyrazine family and its metabolites, was extracted from water and vegetable samples using double-layered magnetic MOF.^149^ The double-layered MOFs were the Fe_3_O_4_-functionalized ZIF-8 coated with a layers of ZIF-69. The composites showed high adsorption, with more than 95% pesticide removal.

## 4 Conclusion and Future Perspectives

The intrinsic tunability of MOFs makes them an appealing choice as a vehicle for controlled release applications as well as environmental cleanup.

For controlled release, the studies have all demonstrated the efficacy of the loaded MOF composite formulation at releasing pesticides into the environment, with comparable and even superior effectiveness to conventional pesticide formulation. The controlled release ability was further enhanced with the modification into composites that would disintegrate when triggered by appropriate stimuli, like pH, microbial enzymes, and sunlight, allowing for a multi-stimuli-controlled release. In addition, multiple studies have shown that the loading of pesticides into the MOFs protects them against photodegradation. Besides delivering pesticides, biocompatible MOFs helped plant growth by supplying micronutrient metal ions as the disintegrate.

Studies on adsorption and extraction of pesticides using MOFs showed promising efficacy, with very high adsorption capacities and good potential for reusability. Different types of strategies have been introduced for the enhancement of adsorption, such as linker defects, composite functionalization, pyrolysis, cationic MOFs. These modifications have contributed by either (i) enhancing the adsorption capacity, by adding an abundance of adsorption sites and/or increasing the surface area or (ii) improving functionality for real-world applications and recyclability. The studies have demonstrated that combination of mechanisms contribute to the adsorption, like electrostatic attraction, π–π stacking, hydrogen bonding, and acid–base interactions. Moreover, kinetic modeling showed the pseudo-second-order model to best describe the adsorption in majority of the studies, indicating that the adsorption primarily relies on chemisorption, and that the adsorption rate depends on the adsorption capacity rather than concentration.

**Scheme 2:**
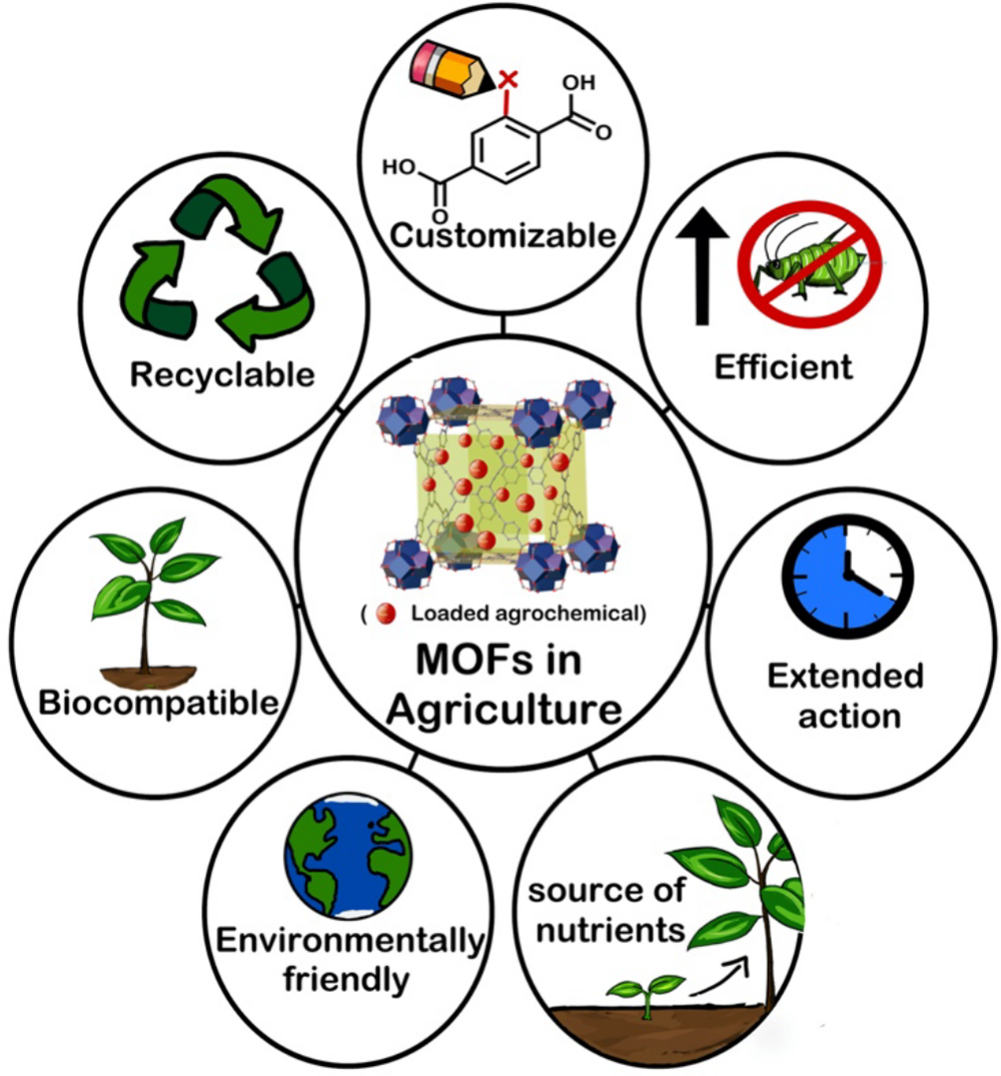
Overview of Different Potential Applications of MOFs in Agriculture

As shown in Scheme 2, MOFs are promising for a range of agricultural applications. The use of MOFs for the extraction and delivery of agrochemicals has several advantages over conventional adsorptive porous materials. The relative high surface area of MOFs as well as their modular nature allow functionalization of the pores resulting in high adsorptive/loading capacities than other porous materials. MOFs are also highly versatile with possible post synthetic modifications, and that allows to engineer the MOFs with selective adsorption of guest molecules. Additionally, decent reusability were demonstrated for the majority of the MOFs studied. Despite the potential advantages of MOFs, there are still some challenges regarding their use in agricultural applications. For example, many MOFs involve the use of toxic metals like Cr and Ni, and with their possible disintegration, these toxic metals can accumulate in the environment. Additionally, the modification of linkers can alter some factors that are essential for incorporating guest molecules, like the surface charge, which in turn, limits the ranges of pH for optimum adsorption/loading conditions and limits their use in real-world applications. Furthermore, the synthesis methods of MOFs are neither cost-effective nor environmentally friendly, often involving many steps and energy intensive. Being a relatively new area of research, there is also limited data available on long-term studies for the effects of MOF and MOF composites in agricultural applications.

Hence, there are several important factors that are needed to be considered to select which MOF/MOF-composite to use for agricultural applications. The MOFs must be biocompatible, posing no risk on crops. They also should possess good relative stability in working conditions, to prevent their contamination and the loss of their efficiency. in addition, other factors, like the interactions of MOFs/MOF-composites with the agrochemical should be taken into consideration, for determining the optimum conditions required.

However, studies utilizing on-field applications are still scarce, and further investigations testing the effect of multiple contaminants on the adsorption efficacy are needed. In addition, the lack of full-scale life-cycle analysis studies on the use of MOFs/MOF composites is necessary to compare with conventional pesticide application/remediation methods to investigate real-world commercial utilization.

Nonetheless, the field of MOFs in agricultural applications is relatively new and currently attracting increasing attention with particular focus on controlled delivery of pesticides. Despite the challenges facing this new application, MOFs have proven to be advantageous and promising for agricultural applications.

## Abbreviations

2,4-D: 2,4-dichlorophenoxyacetic acid
MCPA: 2-methyl-4-chlorophenoxyacetic acid
DDT: dichlorodiphenyltrichloroethane
GABA: γ-aminobutyric acid
MOF: metal–organic framework
HPLC: high-performance liquid chromatography
PDA: polydopamine
CMCS: carboxymethyl chitosan
DNF: dinotefuran
GSH: glutathione
EDTA: ethylenediaminetetraacetic acid
NIR: near-infrared radiation
DMF: dimethylformamide
LC: λ-cyhalothrin
TMX: thiamethoxam
LC_50_: minimum lethal concentration 50
ED_50_: median effective concentration
PCL: polycaprolactone
IMI: imidacloprid
BET: Brunauer–Emmett–Teller
HKUST: Hong Kong Institute for Science and Technology
UiO: University of Oslo
AZOX: azoxystrobin
PMMA: poly(methyl methacrylate)
PVA/ST: poly(vinyl alcohol)/starch
DCl: deuterium chloride
POM: polyoxometalate
BP: black phosphorus
CA: chitosan alginate
BSA: bovine serum albumin
PAN: polyacrylonitrile
CSA: chitosan aerogel
MCPP: mecoprop
MDC: MOF-derived carbon
IL: ionic liquid
TOC: total organic carbon
GO: graphene oxide
IPU: isoproturon
